# The variable association between expression and methylation of estrogen receptors and the survival of patients with different tumors

**DOI:** 10.1002/ctm2.49

**Published:** 2020-06-14

**Authors:** Chao Hu, Yinhua Liu, Shan Jiang, Hongjin Chen, Haojun Xu, Junhong Hu, Congzhu Li, Hongping Xia

**Affiliations:** ^1^ State Key Laboratory of Reproductive Medicine & Department of Pathology in the School of Basic Medical Sciences & The Affiliated Sir Run Run Hospital & Key Laboratory of Antibody Technique of National Health Commission Nanjing Medical University Nanjing China; ^2^ Department of Pathology The First Affiliated Yijishan Hospital of Wannan Medical College & Key Laboratory of Non‐coding RNA Transformation Research of Anhui Higher Education Institutes Wannan Medical College Wuhu China; ^3^ Department of Oncology The First Affiliated Hospital of Chongqing Medical University Chongqing China; ^4^ Department of General Surgery Huaihe Hospital of Henan University Kaifeng China; ^5^ Department of Gynecologic Oncology Cancer Hospital of Shantou University Medical College Shantou Guangdong China

**Keywords:** estrogen receptor, methylation, mRNA, protein, survival

## Abstract

**Background:**

Estrogen receptor (ER) is essential in reproductive development and is also the primary driver of breast cancers. Deregulation of ER may also be involved in tumorigenesis of other organs. To understand the role of ER in different tumor types, pan‐cancer analysis of estrogen receptor alpha (ESR1) and estrogen receptor beta (ESR2) in various tumors and association with patients' survival were conducted using The Cancer Genome Atlas (TCGA) data.

**Results:**

Gene methylation level was evaluated by the mean methylation level of CpG sites in the promoter region. The significant different DNA methylation between tumor and healthy tissues was shown in 10 tumor types for ESR1 and eight tumor types for ESR2. The methylation pattern was also varied across different TCGA tumors. The pan‐cancer analysis showed significantly different mRNA expression of ESR1 in nine tumor types and ESR2 in four tumor types. Survival analysis showed that the effects of ERs expression on survival are diverse in different tumors. The expression of ERs was associated with tumor molecular subtypes and various clinical characteristics. ER correlated genes were mainly enriched in cancer and immune‐related pathways.

**Conclusions:**

Our pan‐cancer analysis data indicated that ERs might be significantly associated with carcinogenesis and progression of some tumors, which may be potential therapeutic targets and prognosis biomarkers.

## Graphical abstract

**Graphical Headlights**

- Deregulation of ERs are involved in tumorigenesis of different organs.
- Different expression/methylation of ESR1/2 were identified in some tumor types.
- The expression/methylation of ESR1/2 were associated with survival of patients.
- ERs and related genes may be potential therapeutic targets or prognosis biomarkers.

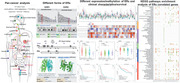

## 1 BACKGROUND

Cancer development is close to hormone level, and hormone level is related to the prognosis and agent insensitivity of some cancer patients. There are three types of steroid hormone receptors, including androgen receptors (ARs), estrogen receptors (ERs), and progesterone receptors (PRs). Emerging evidence showed that steroid hormone receptors mediated signaling plays critical roles in cancer initiation, progression, metastasis, and prognosis as well as sexual dimorphism of some cancers.^1^, ^45^ Estrogen receptor alpha (ERα) and estrogen receptor beta (ERβ) are two distinct ERs, which are encoded by ESR1 (NR3A1) and ESR2 (NR3A2) genes, respectively.^20^ ERs play an important physiological role in reproduction, nervous, endocrine, immune, and cardiovascular systems.^30^ ERs are nuclear hormone receptors and induce transcription elements that further promote tumor growth via binding to regulatory factors. ER signal pathway medicated various diseases, including cancer.

The ER is a critical factor that has been extensively reported in breast cancer. Intratumor heterogeneity of the ER increased the long‐term risk of fatal breast cancer.^23^ ERα is one of the primary drivers of breast cancers, and ER+ cases by immunohistochemistry staining are responsive to endocrine therapies with a better prognosis.^16^ ESR1 mutations are frequently detected in ER+ metastatic breast cancer and may be associated with endocrine therapy resistance.^31^ Besides several hormone‐responsive cancer types, emerging studies showed that ERs might also play an essential role in other cancer types. ERs have the potential to become the prognostic and therapeutic targets for lung cancer.^19^ High ERβ expression in tumor epithelial cells of lung cancer has been reported as a negative prognosticator in females patients.^35^ There are significant gender differences in liver cancer.^43^ Hepatocellular carcinoma (HCC) patients' have increased levels of G‐protein‐coupled estrogen receptor (GPER1) compared with nontumor tissue samples. Estrogen can accelerate hepatocarcinogenesis in male zebrafish, while GPER1 reduced tumor development.^10^ Recent studies revealed a luminal subtype of bladder cancer initiation and progression that exhibited an ER signaling pathway.^15^ ERβ may be a critical target for melanoma^25^ and colorectal cancer prevention.^42^ However, the critical role of ERs signaling in other hormone‐independent human malignancies is poorly understood. Thus, we hypothesized that ESR1 and ESR2 might involve in the progression and prognosis of various cancers. Meanwhile, the expression and methylation difference of ESR1 and ESR2 in tumors and matched healthy tissues should reveal some critical information in the clinic, and in male or female, its expression would be the evident difference.

## 2 MATERIALS AND METHODS

### 2.1 Data resource

All the data used for analyses were downloaded from the Pan‐Cancer Atlas Project (https://gdc.cancer.gov/about-data/publications/pancanatlas), which compares the 33 tumor types profiled by The Cancer Genome Atlas (TCGA). The previous study showed that the overall survival (OS) and progression‐free interval (PFI) are relatively accurate in TCGA data.^24^ We also extracted mRNA, methylation, and molecular protein data of ESR1 and ESR2 provided by Pan‐Cancer Atlas. Normalized ESR1 and ESR2 expression values were transformed by log2(x+1) before subsequent analysis. The methylation beta value was obtained from the HumanMethylation450 platform of TCGA samples. The ESR1 gene region contained 63 methylation CpG sites with 47 sites in the promoter region, and ESR2 included 21 CpG sites with 14 sites in the promoter region in the HumanMethylation450 platform. As the gene expression is strongly associated with DNA methylation in promoter regions, the methylation level of the individual gene was evaluated by the average methylation value of probes in the promoter region. Protein expression data of ESR1 were obtained from the Reverse phase protein array (RPPA) data of the TCGA Pan‐Cancer Atlas.

Microarray expression data were searched and downloaded from the Gene Expression Omnibus (GEO) database (https://www.ncbi.nlm.nih.gov/geo/). We downloaded eight microarrays set from four different cancers, including GSE63514 and GSE63678 of cervical cancer; GSE73360 and GSE74602 of colorectal cancer; GSE76297 of cholangiocarcinoma; GSE87630, GSE112790, and GSE121248 of hepatocellular cancer.

### 2.2 Data analysis and statistical methods

All data analysis and statistics were performed using the statistical package R, version 3.6.1. Gene expression difference between tumor and normal tissues of each cancer type was compared using the Wilcoxon rank‐sum test. The correlation coefficient was calculated using the Spearman method. The association of gene expression with different clinical characteristics of each tumor type was calculated using the Kruskal‐Wallis test. All *P*‐values are two‐sided, and *P*‐values ≤ .05 were considered statistically significant. Gene enrichment analysis was performed using the R *clusterProfiler* package.

We investigated the association between ESR1/2 expression and patient survival by *survival* package of R. The median expression values of ESR1/2 were set as the cut‐off for each tumor type of dividing patients into two groups. The Kaplan‐Meier method was used to compare the OS and PFI between high and low‐ESR1/2 expression groups in each tumor. The *P*‐values were calculated using the log‐rank test. The influence of ESR1/2 expression on other clinical characteristics, such as patients' age, gender, and race, and tumor status, stage, and grade, was also compared. We estimate the survival based on the ESR1/2 expression and different clinic‐pathologic factors using the Cox regression analysis.

## 3 RESULTS

### 3.1 A pan‐cancer analysis of ER molecular level difference in TCGA cancers

To investigate the potential role of ER in human cancers, we performed the pan‐cancer investigation of ER methylation, mRNA, and molecular protein data obtained from TCGA. The necessary information of each TCGA tumor type is reported in Table S1.

**FIGURE 1:**
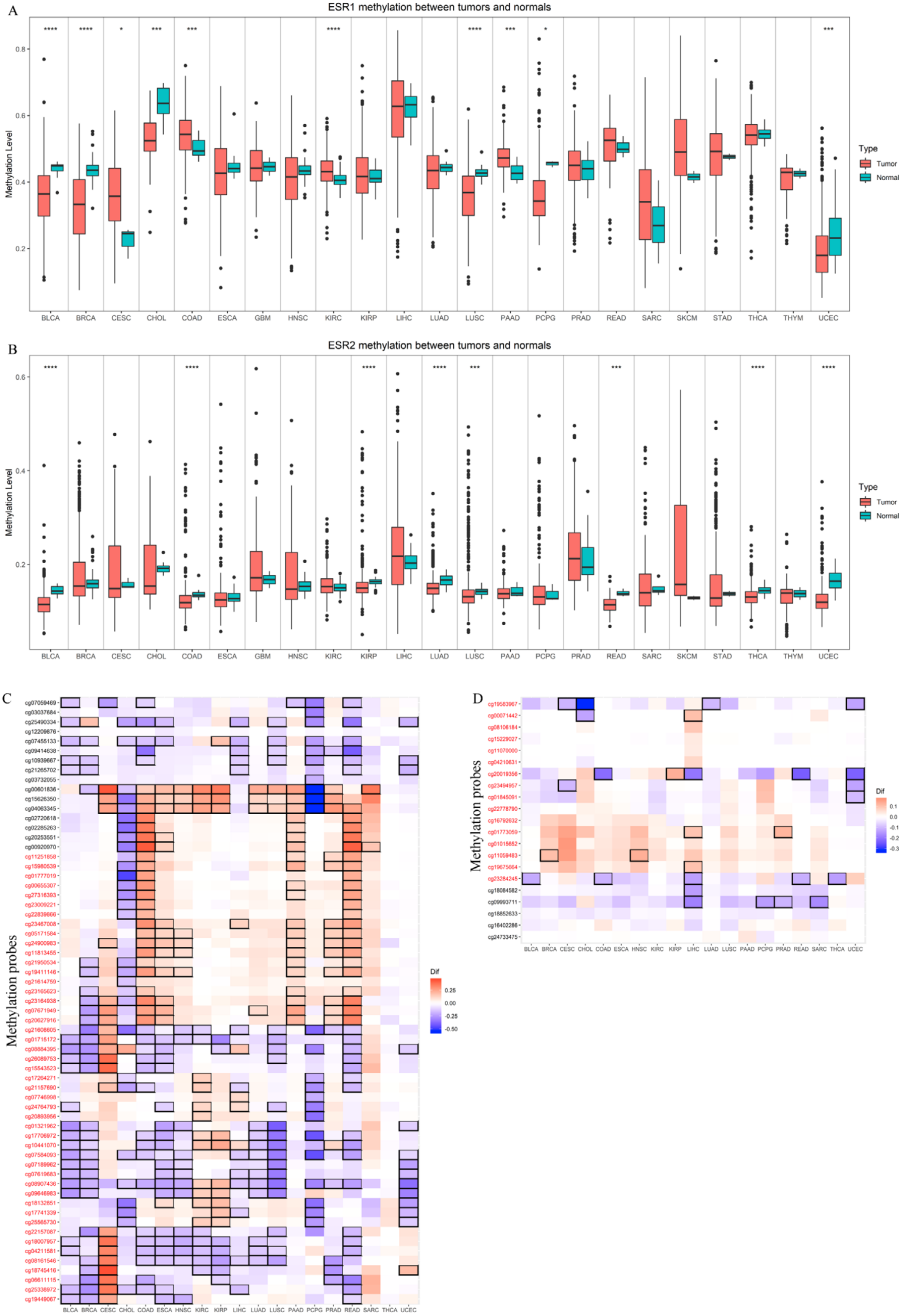
Methylation level of ers in different tumor types. A, B, ESR1 (A) and ESR2 (B) promoter region methylation difference between tumor and adjacent normal tissues in different tumor types; C, D, ESR1 (C) and ESR2 (D) probes methylation difference between tumor and adjacent normal tissues in different tumor types; Asterisks represent statistically significant differences. ^*^ *P*‐value < .05; ^**^ *P*‐value < .01; ^***^ *P*‐value < .001. The color indicates correlation coefficients, and the black border indicates a statistically significant correlation

We extracted the ESR1 and ESR2 DNA methylation levels from the HumanMethylation450 platform of TCGA, including 63 CpG sites in the whole ESR1 region and 21 CpG sites in whole ESR2 region. Methylation is a crucial epigenetic regulation mechanism; the DNA methylation in promoter regions is strongly associated with gene expression and could be a predictor of patients' prognosis.^13^, ^27^ We evaluated ESR1 and ESR2 gene methylation level by calculating the mean methylation level of CpG sites in the gene promoter region of genes (47 probes in ESR1 and 16 probes in ESR2). Six tumor types (including BLCA, BRCA, CHOL, LUSC, PCPG, and UCEC) showed significant lower DNA methylation of ESR1 in tumor tissues than healthy tissues and four tumor types (including CESC, COAD, KIRC, and PAAD) significant higher DNA methylation of ESR1 in tumor tissues (Figure 1A). ESR2 showed a low methylation level in eight tumor types, including BLCA, COAC, KIRP, LUAD, LUSC, READ, THCA, and UCEC (Figure 1B). According to the methylation differences of different CpG sites, we found that although CpG sites methylation difference did not remain consistent in different tumors, methylation patterns were similar in some tumors such as COAD, READ, and ESCA (Figure 1C,D).

**FIGURE 2:**
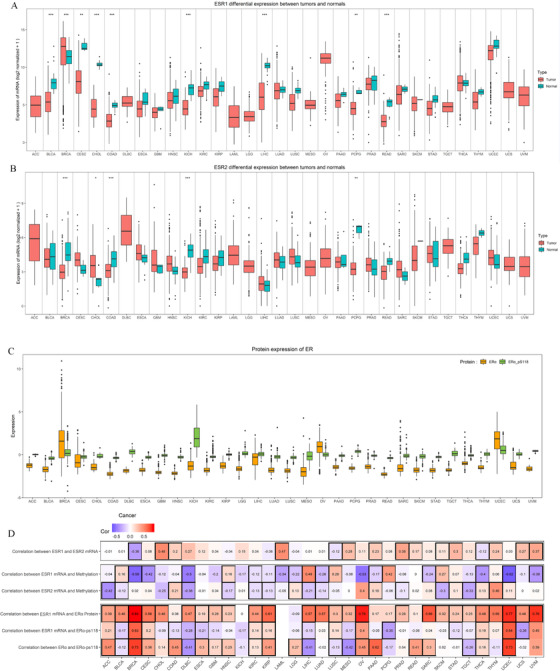
Expression and correlation of ERs in different tumor types. A, B, ESR1 (A) and ESR2 (B) mRNA expression between tumor and normal tissues in different cancer types. Asterisks represent statistically significant differences. ^*^ *P*‐value < .05; ^**^ *P*‐value < .01; ^***^ *P*‐value < .001. C, Expression of ESR1 coded protein in different tumor types. D, Heatmap of correlation among ESR1 and ESR2 mRNA expression, methylation level, and protein ERα and ERα‐pS118 expression in different tumor types. The color indicates correlation coefficients, and the black border indicates a statistically significant correlation

We then compared ESR1 and ESR2 mRNA expression levels difference in 23 cancer types with both tumors and adjacent normal tissues. The significantly different mRNA expression level was shown in nine tumor types (*P*‐value < .05 and an absolute log_2_ fold change  >  1). High expression of ESR1 was ascertained in tumor tissues compared with normal tissues in BRCA, while other tumors showed low expression, including BLCA, COAD, CESC, CHOL, KICH, LIHC, PCPG, and READ (Figure 2A). The ESR2 expression was lower in tumor tissues than healthy tissues in BRCA, COAD, KICH, and PCPG, and higher in CHOL (Figure 2B). We got similar results by comparing the mRNA expression of ESR1and ESR2 between exactly matched tumor and normal tissues from the same patients (Figure S1). Next, we obtained protein expression data of different tumors from TCGA. The RPPA data provide relative protein expression of the ESR1 gene, which includes two proteins ERα and ERα‐pS118. The expression of ERα was lower than ERα‐pS118 in most tumors, except BRCA, OV, and UCEC (Figure 2C).

Furthermore, we calculated the correlations between ESR1 and ESR2 mRNA expression, correlations between mRNA and methylation level, and correlations between mRNA and protein expression. The analysis revealed that correlation coefficients are diverse across different tumors (Figure 2D; Figure S2). Other sex hormone receptors, not just estrogen receptors, also played an important role in tumors, such as androgen receptor (AR) and progesterone receptor (PGR). According to the androgen receptor, progesterone receptor, estrogen receptor expression, and correlation analysis between these genes, we observed that the expression levels of different hormone receptors in different tumor tissues varied greatly and the correlation between genes also varied in different tumors, suggest that the sex hormone receptors signals may act the independent or synergistic role in different tumors (Figure S3A). Interestingly, a high correlation was observed among AR, ESR1, and PGR genes in gastrointestinal tumors (COAD, READ, and STAD) as they may play a synergistic role in these tumors (Figure S3B).

### 3.2 ER expression of GEO data set in different cancers

**FIGURE 3:**
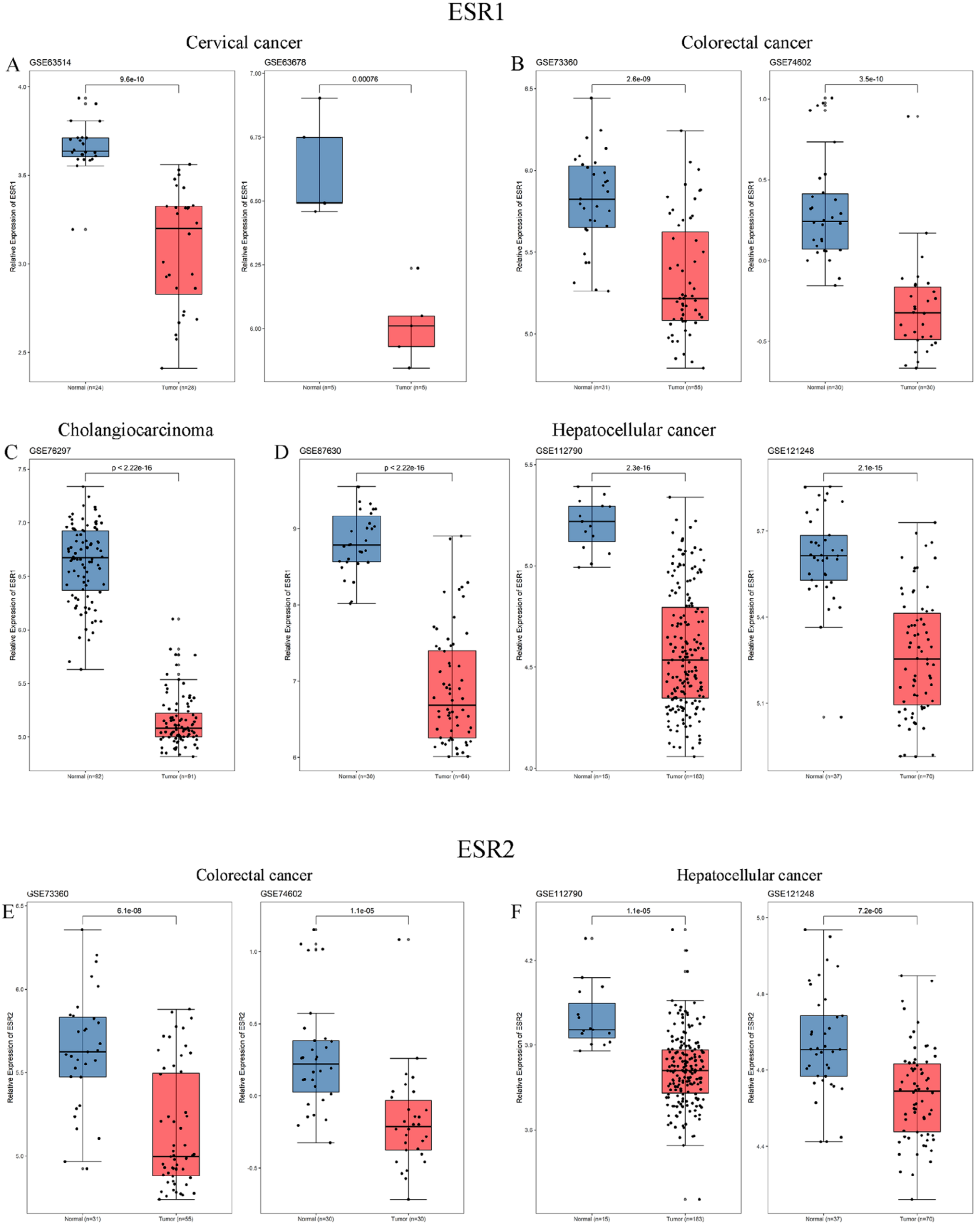
mRNA expression of estrogen receptors in GEO microarray datasets. A, The expression between tumor and normal in cervical cancer of GSE63514 and GSE63678 datasets; B, Expression between tumor and normal in cholangiocarcinoma of GSE76297 dataset; C, Expression between tumor and normal in colorectal cancer of GSE73360 and GSE74602; D, Expression between tumor and normal in hepatocellular carcinoma of GSE87630, GSE112790, and GSE121248 datasets. E, The expression between tumor and normal in colorectal cancer of GSE73360 and GSE74602; F, Expression between tumor and normal in hepatocellular carcinoma of GSE87630, GSE112790, and GSE121248 datasets. *P*‐value was marked in the upper part of each dataset figure, and every dataset showed significant statistical significance

To confirm the estrogen receptor expression result, we observed in TCGA data. We extracted estrogen receptor genes expression level of different tumor data sets in the GEO database. We compared the expression of estrogen receptor gene in eight microarray expression datasets of four tumors (Cervical cancer: GSE63514 and GSE63678; Colorectal cancer: GSE73360 and GSE74602; Cholangiocarcinoma: GSE76297; Hepatocellular cancer: GSE87630, GSE112790 and GSE121248). We observed low ESR1 expression levels from eight datasets in four tumor types, consistent with ESR1 expression level in TCGA tumors (Figure 3A–D). Moreover, the low ESR2 expression level was confirmed from four datasets in colorectal cancer and hepatocellular cancer (Figure 3E,F).

### 3.3 Association of ER expression with molecular subtypes and clinical variables

**FIGURE 4:**
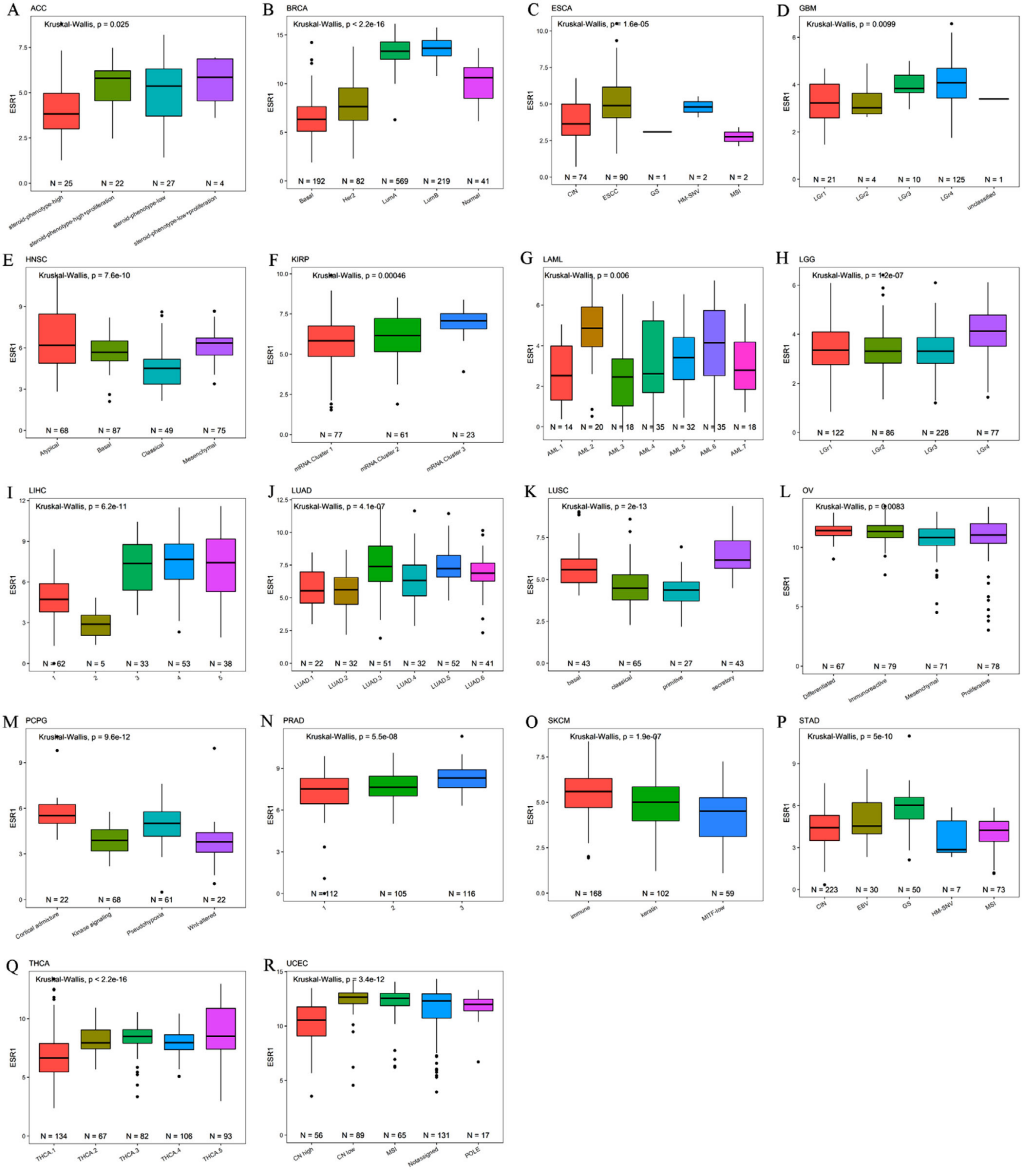
The association of ESR1 mRNA level with tumor molecular subtypes in TCGA cancers. The expression of ESR1 is significantly different among molecular subtypes in (A) ACC, (B) BRCA, (C) ESCA, (D) GBM, (E) HNSC, (F) KIRP, (G) LAML, (H) LGG, (I) LIHC, (J) LUAD, (K) LUSC, (L) OV, (M) PCPG, (N) PRAD, (O) SKCM, (P) STAD, (Q) THCA, and (R) UCEC. We only show the tumor types with significant statistical significance. The *P*‐value was calculated by the Kruskal‐Wallis test and marked in the upper right corner of each figure

**FIGURE 5:**
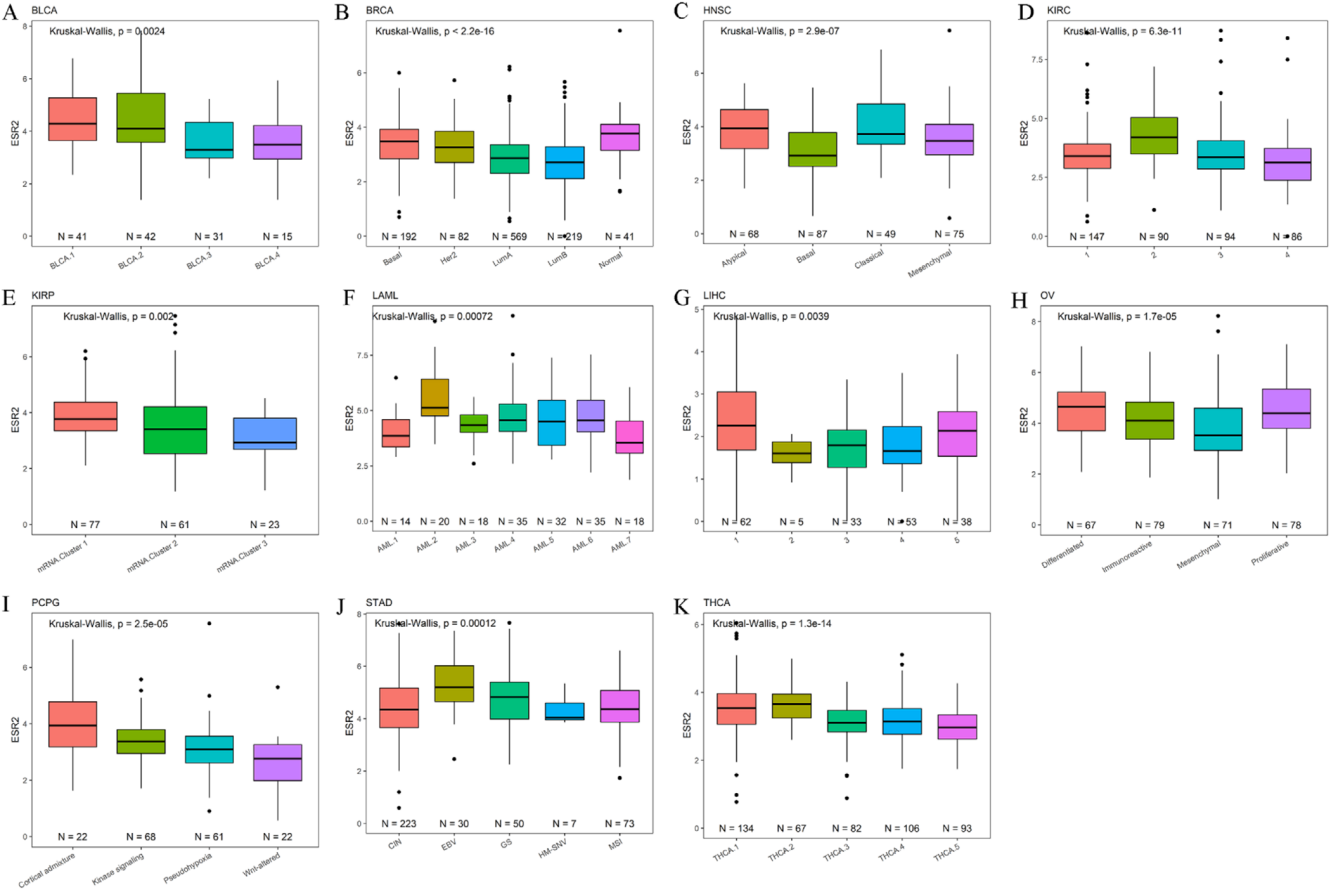
The association of ESR2 mRNA expression with tumor molecular subtypes in TCGA cancers. The expression of ESR2 is significantly different among molecular subtypes in (A) BLCA, (B) BRCA, (C) HNSC, (D) KIRC, (E) KIRP, (F) LAML, (G) LIHC, (H) OV, (I) PCPG, (J) STAD, and (K) THCA. We only show the tumor types with significant statistical significance. The *P*‐value was calculated by the Kruskal‐Wallis test and marked in the upper right corner of each figure

To understand the effect of the ER in different tumor types, we analyzed the correlation of ESR1and ESR2 mRNA level with the molecular subtype of 24 TCGA tumor types. We obtained molecular subtype data for different TCGA tumors. Eighteen of 24 tumors, including ACC, BRCA, ESCA, GBM, LGG, HNSC, KIRP, LAML, LUAD, LUSC, LIHC, OV, PRAD, PCPG, STAD, SKCM, THCA, and UCEC, showed significant ESR1 mRNA expression differences across different molecular subtypes (Figure 4). Eleven tumors, including BLCA, BRCA, HNSC, KIRP, KIRC, LIHC, LAML, OV, STAD, PCPG, and THCA, showed significant ESR2 mRNA expression differences across different molecular subtypes (Figure 5). Further analysis revealed some common or unique characteristics between molecules across different tumors. ESR1 expression was correlated with tissue‐specific genes in ACC and THCA, while ESR1 and ESR2 were correlated with cell adhesion‐related genes such as claudin‐7 (CLDN7; Figure S4). In LIHC and UCEC, ESR1 expression was associated with proliferation and mitotic genes, and correlation analysis showed that ESR1 was negatively correlated with proliferation and cell cycle genes in most tumor types (Figure S5). We also noticed that IDH‐mutant or IDH‐like LIHC and PRAD samples belonged to subtypes with low ESR1 expression, while high ESR1 subtype was exclusively IDH‐wild‐type in glioma (GBM and LGG), and the expression of ESR1 was lower in IDH1 mutated samples (Figure S6). For each cancer type, the expression of ESR1 and ESR2 across different patients’ clinical variables were analyzed. Moreover, we found that the expression of ESR1 and ESR2 were associated with clinical variables such as gender, age, race, grade, stage, and tumor status (Figures S7 and S8).

### 3.4 Association of ER expression with the survival of TCGA patients

**FIGURE 6:**
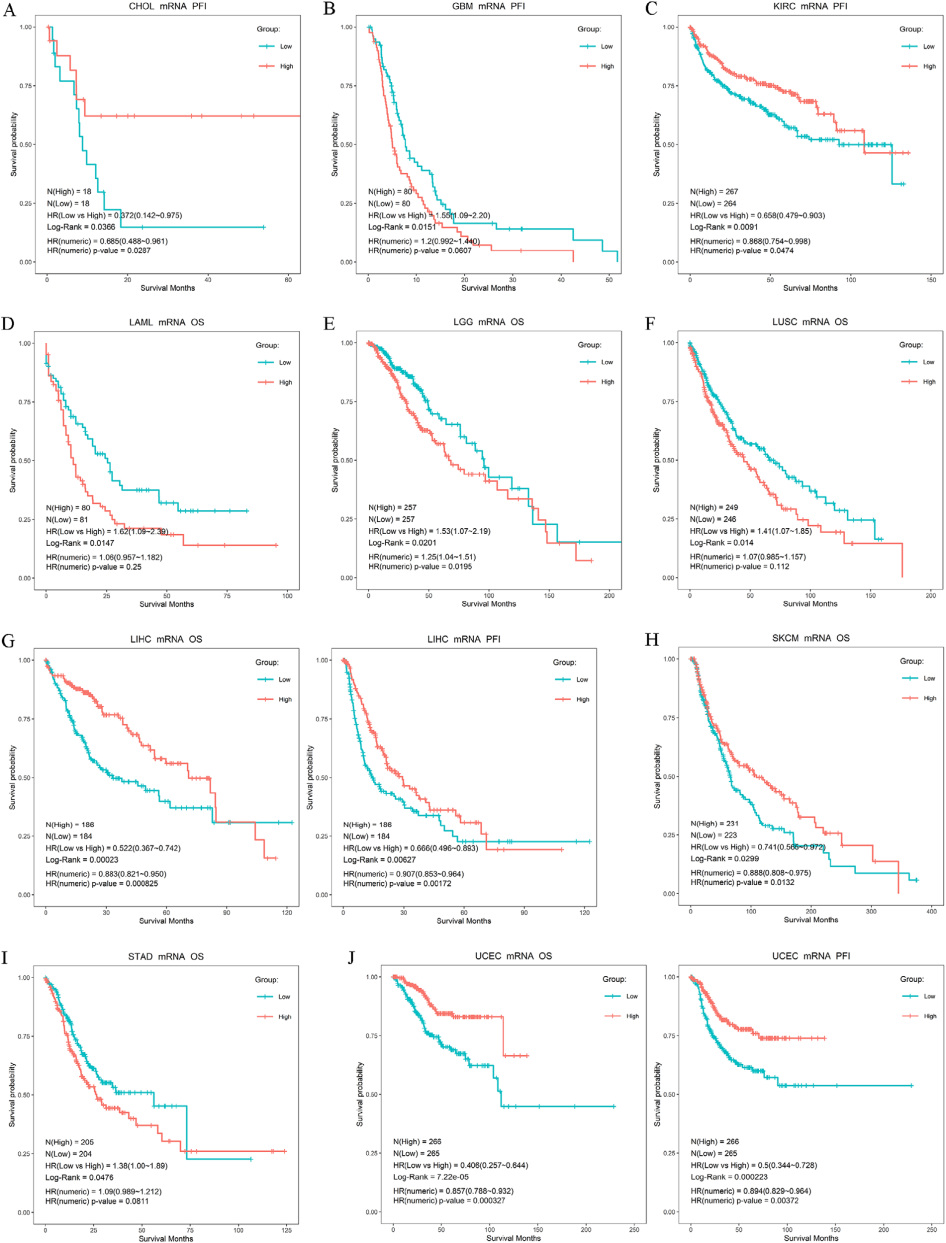
The association of ESR1 expression with patients' survival in TCGA cancers. Statistically significant survival difference (log‐rank p‐value < 0.05) between high and low ESR1 group were found in (A) CHOL, (B) GBM, (C) KIRC, (D) LAML, (E) LGG, (F) LUSC, (G) LIHC, (H) SKCM, (I) STAD, (J) UCEC

**FIGURE 7:**
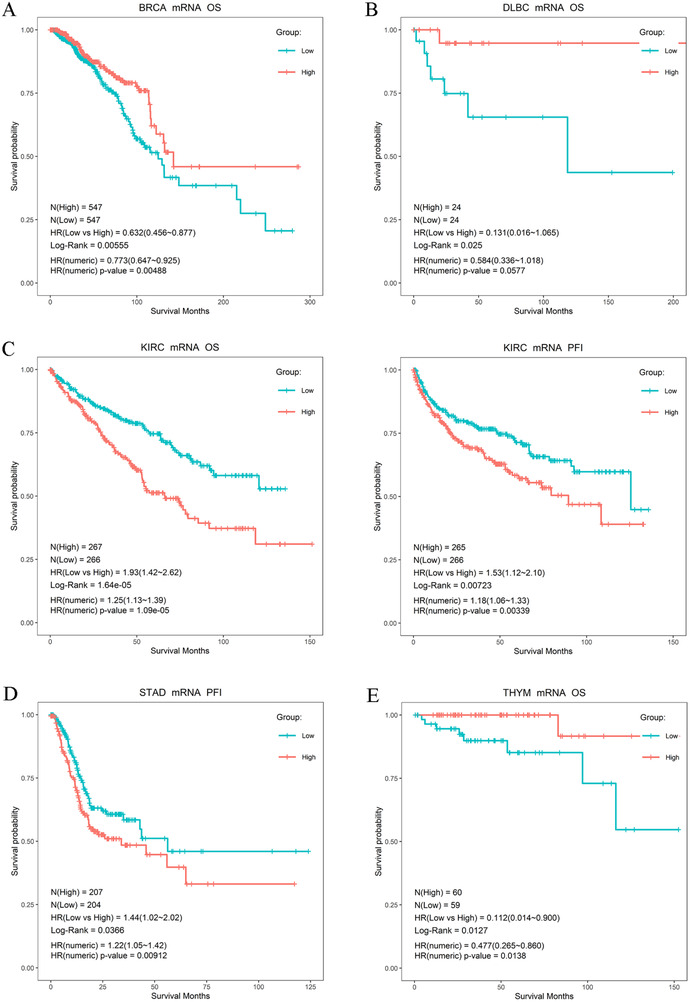
The association of ESR2 expression with patients' survival in TCGA cancers. Statistically significant survival difference (log‐rank p‐value < 0.05) between high and low ESR1 group were found in (A) BRCA, (B) DLBC, (C) KIRC, (D) STAD, (E) UCEC

To study the effect of ER expression on survival in different cancers, we divided patients into high‐ and low‐group using the median expression value of ESR1/2 mRNA as the cut‐off. As shown in Figure 6A–J, the overall survival rate of ESR1 mRNA level was a statistically significant difference in LAML, LGG, LUSC, LIHC, SKCM, STAD, UCEC, and the progression‐free interval rate was a statistically significant difference in CHOL, GBM, KIRC, LIHC, and UCEC. The overall survival rate of ESR2 mRNA level was a statistically significant difference in BRCA, DLBA, KIRC, THYM, and the progression‐free interval rate was a statistically significant difference in KIRC and STAD (Figure 7A–E). Notably, in GBM, LAML, LGG, LUSC, and STAD individuals, lower expression of ESR1 showed a better survival rate than a higher expression of ESR1 (Figures 6B, 6D–F, and 6I). Besides, compared with low ESR2 mRNA level, high mRNA level ESR2 was ascertained worse prognosis in KIRC and STAD (Figure 7C,D). These results suggest that ESR1 and ESR2 participated in the progression and development of cancer treatment. However, the role of ESR1 or ESR2 had a considerable difference in diverse tumor types.

Survival differences between high‐ and low‐methylation of ESR1/2 group patients were compared. Moreover, we found that the overall and progression‐free survival was longer in the high ESR1 methylation group in BLCA, BRCA, LAML, LGG, and STAD, but shorter in KIRC and KIRP (Figure S9A–G). In the ESR2 high‐methylation group, the overall and progression‐free survival was significantly longer in LGG and STAD (Figure S9H,I).

Survival analysis of the TCGA database by the level of ERα and ERα‐pS118 was also analyzed. In PRAD was higher expression of ERα associated with poor survival while ACC, KIRC, KIRP, LGG, LIHC, SKCM, and UCEC were a higher expression of ERα associated with better survival (Figure S10). Meanwhile, ERα‐pS118 higher expression showed worse survival in HNSC (Figure S5F). In other cancer types, including BLCA, KIRP, KICH, and UCEC, higher ERα‐pS118 level revealed greater survival than lower level (Figure S11). Overall, these data demonstrated that higher expression of ERα or over‐phosphorylation of ERα in the S118 site should protect and prolong tumor patient life, except for a little particular type.

### 3.5 The univariate and multivariate analysis of ERs expression in different tumor types

We next further performed the cox regression analysis of ESR1/2 mRNA expression, DNA methylation and ERs protein expression levels in each tumor type. Continuous gene expression data were used in univariate analysis. Different clinical characteristics, such as age, gender, race, tumor status, stage, and grade, were considered in multivariate analysis. As a result, as shown, after adjusting the age, gender, and race of patients, most tumors still showed a significant association with patients' prognosis. However, when we further adjust the tumor stage/grade/status of the patients, only LIHC and MESO showed significant survival association with ESR1 mRNA expression (Table S2), while BRCA, KICH, KIRP, LGG, and PAAD patients survival significant association with ESR2 mRNA expression (Table S3). Similar results were obtained in methylation and protein data (Tables S4–S7).

### 3.6 Pathways enrichment analysis of ERs expression significantly correlated genes

**FIGURE 8:**
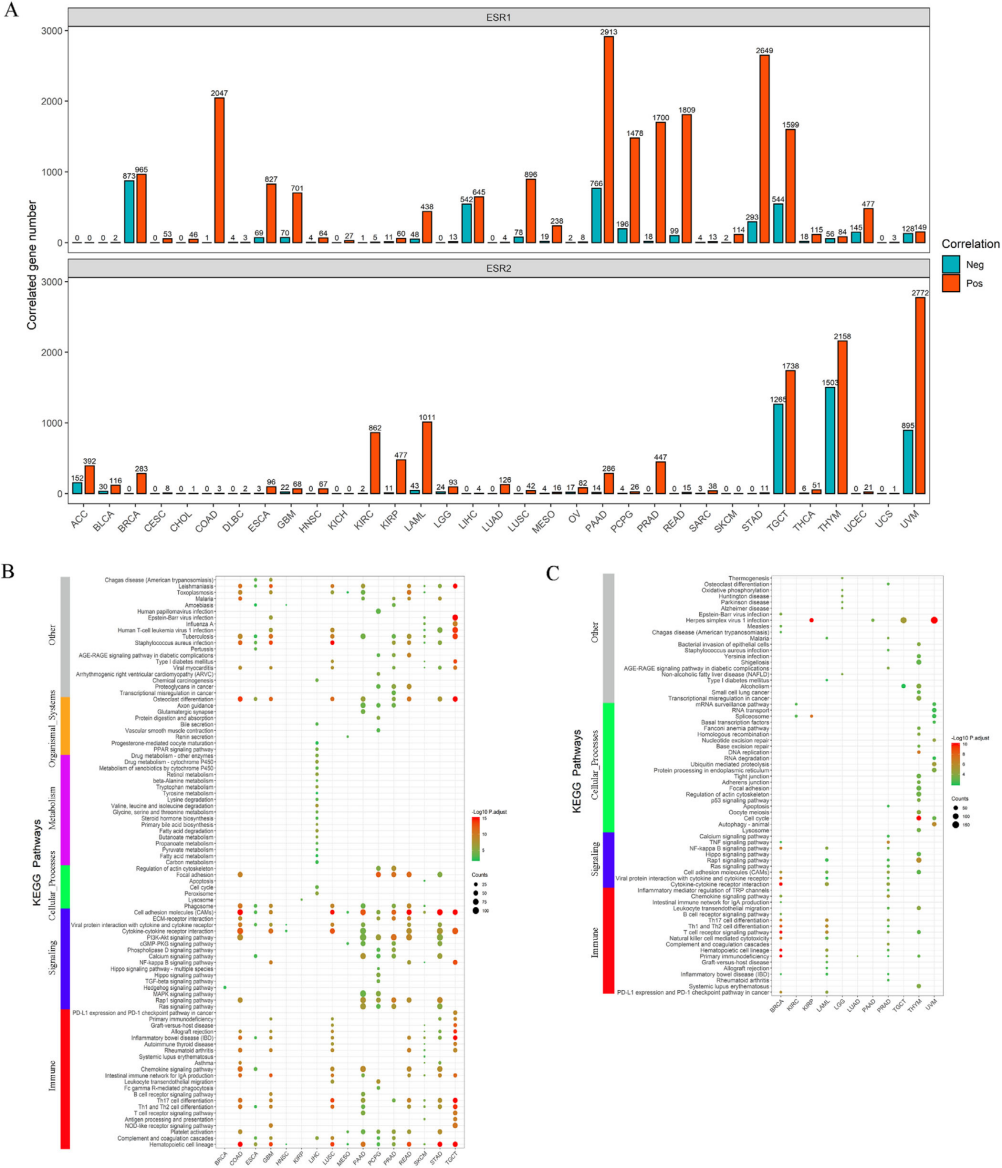
KEGG pathways enrichment analysis of ERs significantly correlated genes. (A) The number of ESR1 significantly correlated genes (top) and ESR2 significantly correlated genes (bottom) in each TCGA tumor. The red bar indicates a positive correlation and blue bar indicates a negative correlation. (B)Enriched KEGG pathways of ESR1 correlated genes. (C) Enriched KEGG pathways of ESR2 correlated genes. The color of the dot indicates the adjusted p‐value, and the size indicates the number of genes enriched in the pathway

To investigate the role of ERs in different tumors, correlation analysis was applied for each TCGA cancer types using a spearman's method and the correlation coefficients between ESR1/2 and other genes were calculated. The genes with the absolute correlation coefficient value greater than 0.4 and the adjusted *P*‐value < .05 were considered to be ER significantly correlated genes. The number of ERs significantly correlated genes varies considerably in different tumor types (from 0 to 3679 for ESR1 and from 0 to 3667 for ESR2; Figure 8A). The ER significantly correlated genes of each tumor type were used for the pathway enrichment analysis. The KEGG enrichment result showed that ESR1 and ESR2 significantly correlated genes were mainly enriched in immune response and tumor‐related cellular signaling pathways in most cancer types (Figure 8B,C). GO enrichment results showed that ESR1 and ESR2 relate biological processes include immune cell activity and cellular RNA processing (Figure S12).

## 4 DISCUSSION

Our study investigated the role of estrogen receptors in different tumors and found that the expression and methylation were significant differences between tumor and normal tissues and associated with patient's survival in many cancers. The cellular biological processes suggest a negative correlation between DNA methylation and mRNA expression, a positive correlation between mRNA and protein expression, as we observed in most cancers. However, this relationship was not found in some tumors. These result potential showed that the estrogen receptor expression in tumor cells and the development of cancer is complex and complicated, and control of not one gene.

ESR1 and ESR2 expression have been investigated in many cancer types. Recent studies have verified tissues ESR1 mutations in most tumor patients, especially with metastatic breast cancer, and some of them to activate the estrogen‐independent receptor,^18^, ^39^, ^40^ whereas ESR1 and ESR2 expression not only express in breast cancer but also have been shown in other cancer types.^10^, ^15^, ^25^, ^42^ Thus, using the TCGA database, we analyze ESR1 and ESR2 mRNA expression in tumor tissues. A significant expression difference was observed in many tumor types across most human tissues and organs and verified in other microarray sets. Interestingly, even no significant differential ESR1 and ESR2 expression were found in many carcinomas. The overall or progression‐free survival was significantly different between high and low expression patients. However, we noticed that the estrogen receptors expression varies significantly among tumor subtypes even in the same tumor type and the associated with the subtypes features, the association was also demonstrated by the significant correlation between estrogen receptor and key genes in the cell cycle, cell differentiation, and junction. As previously reported, the low ESR1 expression was related to high differentiation, high cell adhesion genes, and low immune response genes expression in adrenocortical and thyroid carcinoma.^4^, ^46^ In contrast, low ESR1 expression and high ESR2 expression subtypes present proliferative phenotype in hepatocellular cancer and mitotic phenotypes in endometrial carcinoma.^6^, ^7^ Moreover, the low ESR1 expression subtypes enriched IDH1 mutations in glioma, liver cancer, and prostate adenocarcinoma.^5^, ^7^, ^8^ Analysis of clinicopathological information also indicated a significant correlation between estrogen receptor expression and tumor stage, grade, and status. Since tumor subtypes are strongly associated with tumor malignancy, tumor progression, and patient prognosis, the expression varies in different subtypes and pathological factors suggest that estrogen receptor may be associated with tumor development.

ER methylation has previously been reported to be associated with the progression and prognosis of female tumors. Promoter methylation of ESR1 in breast cancer was related to worse overall survival and associated with a lack of response to endocrine treatment.^26^, ^33^ Both primary tumors and paired ctDNA detected methylated ESR1 and the presence of ESR1 methylation correlated with better clinical outcome in ovarian cancer.^14^ Methylation of the ESR1 promoter correlated with tumor grade, while unmethylated ESR1 predicted for chemoradiation resistance in cervical carcinoma.^21^, ^36^ Our study showed the ESR1 methylation difference and its association with survival in BLCA, BRCA, and KIRC. Both ESR1 and ESR2 showed the correlation between promoter methylation and survival in LGG and STAD.

ERα has been shown to play an essential role in different organ systems during human physiological development.^3^ The phosphorylation of ERα further activates the hormone signal pathway and then unique coactivator complexes to specific genes.^2^ ERα expresses in different carcinoma tissues. Shrivastav et al showed that the p‐S118, p‐S167, and p‐S282 of the ERα were positively correlated with breast cancer.^34^ Another study showed that hypoxia‐induced phosphorylation of estrogen receptor at serine 118.^29^ Therefore, to dig deeper into the function of phosphorylation of ERα, we investigated the phosphorylation site of S118. In BRCA, OV, and UCEC tumors that occur only or predominantly in women, the expression of ERα‐pS118 is lower than the expression of ERα. That means ERα‐pS118 or ERα may play a different role in the three tumors. Analyzed the different tumor forms survival rate of high or low expression of ERα and ERα‐pS118, we found KIRP and UCEC is the only cancer type that shows the similar tendency both ERα and ERα‐pS118.

Univariate and multivariate COX analysis showed that the relationship between estrogen receptor status and the tumor was independent of age, sex, and race as the overall and progression‐free survival still with a significant difference after we exclude the effect of these factors. After further adjust the effect of tumor‐related pathological factors, the ESR1 mRNA expression independent associated with survival in LIHC and MESO and ESR2 independent associated with survival in BRCA, KICH, KIRP, LGG, and PAAD. Most previous studies reported estrogen receptors as a prognostic marker for hormone‐related tumors, such as ESR1 in thyroid carcinoma, and ESR1 and ESR2 in ovarian and breast cancer.^9^, ^11^, ^12^, ^44^ Our study indicates the potential prognostic significance of estrogen receptors in non‐hormonal tumors. As the prognostic significance of ESR1 in an eight genes assessment model in liver cancer, and ER‐β expression in colorectal cancer and ESR2 polymorphisms in advanced gastric cancer.^17^, ^37^, ^38^ The pathway enrichment analysis result showed that ESR1 and ESR2 correlated genes enriched in some immune response and immune cell activity pathways indicated an essential relationship between estrogen receptor and tumor immunity in many cancer types. Estrogen receptor signaling plays an essential physiological role in the immune system, as well as pathological roles in cancer by regulating innate immune signaling pathways and myeloid cell development.^22^, ^30^ Estrogen receptor signaling decreased proliferative capacity and oncodriver expression in melanoma and rendered melanoma cells more vulnerable to immunotherapy.^28^ As an important biomarker in breast cancer, the ER is not only closely related to tumor‐related intracellular signaling activity but also related to tumor‐infiltrating immune cells such as macrophages, neutrophils, dendritic cells, natural killer cells, and B/T cells.^32^ Estrogen receptor knockout enhanced immune cell infiltration and liver tumorigenesis in the mouse tumor model.^41^ While our study found the correlation between estrogen receptors and immune cell differentiation, immune cell signaling, and inflammation pathways in multiple tumors, target estrogen receptor in combination with immunotherapy may potentially benefit patients.

## 5 CONCLUSION

Overall, our findings revealed DNA methylation and mRNA expression of ESR1 and ESR2, proteins expression of ESR1 in different tumor tissues, and ESR1 and ESR2 participated in some critical cancer development and progression as they associated with tumor subtypes, pathological features, and patients' survival. This pan‐cancer analysis work showed that the expression and methylation of ESR genes are significantly associated with overall survival or progression‐free survival of some tumor types, which may suggest that ESR genes are potential prognosis markers of these tumors. Interestingly, we found that ER signaling may affect tumor immune response and significantly associated with patient's survival. These results suggest that therapies targeting ESR signaling may be beneficial to patients with ER‐associated tumors or tumor subtypes. Further studies are needed to systemic reveal the complex ESR‐mechanism of various cancer cells as well as tumor microenvironment changes during cancer occurrence and progression.

## AUTHORS' CONTRIBUTIONS

CH and HX designed the project and developed the computational method for analysis. CH, HC and HX drafted and revised the manuscript. YL, SJ, HX, CL and JH help some analysis and provide some funding support and constructive suggestions to improve this manuscript. All authors read and approved the final manuscript.

## ETHICS APPROVAL AND CONSENT TO PARTICIPATE

Not applicable.

## CONSENT FOR PUBLICATION

Not applicable.

## COMPETING INTERESTS

There are no conflicts of interest.

## FUNDING

This work was supported by grants from the National Young 1000 Talents Program of China, Jiangsu Province Education Department Grant, Jiangsu Province “Innovative and Entrepreneurial Team” and “Innovative and Entrepreneurial Talent” Grant, Chinese Society of Clinical Oncology Research Foundation and Southeast University‐Nanjing Medical University Cooperative Research Project, and Key research and promotion projects of Henan Province (NO.202102310094), Wu Jieping Medical Foundation of Clinical Research Special Fund (NO.320.2710.1836), and Funding of "Peak" Training Program for Scientific Research of Yijishan Hospital, Wannan Medical College (No.GF2019G19).

## Supporting information

Supporting information

## Data Availability

The data we used for all analyses were obtained from TCGA (The Cancer Genome Atlas) Pan‐Cancer Atlas Project (https://gdc.cancer.gov/about-data/publications/pancanatlas).
