# Supplementary material for: The variable association between expression and methylation of estrogen receptors and the survival of patients with different tumors
Source: Clin Transl Med. 2020 Jun 14;10(2):e49. doi: 10.1002/ctm2.49 (PMC7403838; doi:10.1002/ctm2.49)
Supplement: Supplementary file 1 — Supporting information [file CTM2-10-e49-s001.docx]

**Supporting information**

**The variable association between expression and methylation of estrogen receptors and the survival of patients with different tumors**

Chao Hu^1,#^, Yinhua Liu^2,#^, Shan Jiang^3,#^, Hongjin Chen^1^, Haojun Xu^1^, Junhong Hu^4,*^, Congzhu Li^5,*^, Hongping Xia^1-5,*^

^1^State Key Laboratory of Reproductive Medicine & Department of Pathology in the School of Basic Medical Sciences & The Affiliated Sir Run Run Hospital & Key Laboratory of Antibody Technique of National Health Commission, Nanjing Medical University, Nanjing 211166, China.

^2^Department of Pathology, The First Affiliated Yijishan Hospital of Wannan Medical College & Key Laboratory of Non-coding RNA Transformation Research of Anhui Higher Education Institutes, Wannan Medical College, Wuhu 241002, China

^3^Department of Oncology, First Affiliated Hospital of Chongqing Medical University, Chongqing 400016, China

^4^Department of General Surgery, Huaihe Hospital of Henan University, Kaifeng 475000, China.

^5^Department of Gynecologic Oncology，Cancer Hospital of Shantou University Medical College，Shantou，Guangdong 515000，China

**Running title:** Pan-cancer analysis of estrogen receptors

^#^ These authors contributed equally to this work

**^*^Correspondence to** Hongping Xia, Department of Pathology, School of Basic Medical Sciences & The Affiliated Sir Run Run Hospital, Nanjing Medical University, Nanjing 21116, China. E-mail: xiahongping@njmu.edu.cn (H.X.); OR 873872298@qq.com (C.L.); OR hujhhnuniver@126.com (J.H.).

**Supplementary tables:**

| **Table S1 Tumor names and sample numbers TCGA cohort.** | | | | | |
| --- | --- | --- | --- | --- | --- |
| **Disease Name** | **Cancer Type** | **Gender (Female\|Male)** | **mRNA (N\|T)** | **Methylation (N\|T)** | **RPPA (T)** |
| Adrenocortical carcinoma | ACC | 60\|32 | 0\|79 | 0\|79 | 46 |
| Bladder urothelial carcinoma | BLCA | 108\|304 | 19\|408 | 21\|416 | 344 |
| Breast invasive carcinoma | BRCA | 1085\|12 | 114\|1103 | 87\|787 | 892 |
| Cervical and endocervical cancers | CESC | 307\|0 | 3\|306 | 3\|308 | 173 |
| Cholangiocarcinoma | CHOL | 25\|20 | 9\|36 | 9\|36 | 30 |
| Colon adenocarcinoma | COAD | 216\|243 | 41\|453 | 34\|296 | 363 |
| Lymphoid Neoplasm Diffuse Large B-cell Lymphoma | DLBC | 26\|22 | 0\|48 | 0\|48 | 33 |
| Esophageal carcinoma | ESCA | 27\|158 | 11\|185 | 15\|184 | 126 |
| Glioblastoma multiforme | GBM | 230\|366 | 5\|169 | 2\|152 | 244 |
| Head and Neck squamous cell carcinoma | HNSC | 142\|386 | 44\|522 | 50\|525 | 212 |
| Kidney Chromophobe | KICH | 51\|62 | 25\|66 | 0\|65 | 63 |
| Kidney renal clear cell carcinoma | KIRC | 191\|346 | 72\|534 | 157\|315 | 478 |
| Kidney renal papillary cell carcinoma | KIRP | 77\|214 | 32\|291 | 43\|272 | 216 |
| Acute Myeloid Leukemia | LAML | 91\|109 | 0\|173 | 0\|194 | 0 |
| Brain Lower Grade Glioma | LGG | 230\|285 | 0\|534 | 0\|532 | 435 |
| Liver hepatocellular carcinoma | LIHC | 122\|255 | 50\|374 | 50\|377 | 184 |
| Lung adenocarcinoma | LUAD | 280\|242 | 59\|517 | 30\|461 | 365 |
| Lung squamous cell carcinoma | LUSC | 131\|373 | 51\|502 | 41\|364 | 328 |
| Mesothelioma | MESO | 16\|71 | 0\|87 | 0\|87 | 63 |
| Ovarian serous cystadenocarcinoma | OV | 587\|0 | 0\|309 | 0\|10 | 436 |
| Pancreatic adenocarcinoma | PAAD | 83\|102 | 4\|179 | 10\|184 | 123 |
| Pheochromocytoma and Paraganglioma | PCPG | 101\|78 | 3\|184 | 3\|183 | 82 |
| Prostate adenocarcinoma | PRAD | 0\|500 | 52\|498 | 50\|496 | 352 |
| Rectum adenocarcinoma | READ | 78\|92 | 10\|161 | 7\|94 | 132 |
| Sarcoma | SARC | 142\|119 | 2\|263 | 4\|261 | 226 |
| Skin Cutaneous Melanoma | SKCM | 180\|290 | 1\|472 | 2\|473 | 355 |
| Stomach adenocarcinoma | STAD | 158\|285 | 35\|415 | 2\|393 | 357 |
| Testicular Germ Cell Tumors | TGCT | 0\|134 | 0\|156 | 0\|155 | 122 |
| Thyroid carcinoma | THCA | 371\|136 | 59\|513 | 56\|511 | 379 |
| Thymoma | THYM | 60\|64 | 2\|120 | 2\|124 | 90 |
| Uterine Corpus Endometrial Carcinoma | UCEC | 548\|0 | 34\|533 | 45\|422 | 440 |
| Uterine Carcinosarcoma | UCS | 57\|0 | 0\|57 | 0\|57 | 48 |
| Uveal Melanoma | UVM | 35\|45 | 0\|80 | 0\|80 | 12 |

| \| **Table S2 Survival association with ESR1 mRNA expression using Cox regression** \| \| \| \| \| \| \| \| --- \| --- \| --- \| --- \| --- \| --- \| --- \| \| **Cancer** \| **Overall Survival** \| \| \| **Progression-Free Interval** \| \| \| \| **univariate COX** \| **multivariate COX (age/gender/race)** \| **multivariate COX (all)** \| **univariate COX** \| **multivariate COX (age/gender/race)** \| **multivariate COX (all)** \| \| CHOL \| _ \| _ \| _ \| **0.68 (0.49-0.96) 0.03** \| **0.64 (0.45-0.93) 0.02** \| _ \| \| HNSC \| **0.9 (0.83-0.97) 0.01** \| **0.91 (0.84-0.99) 0.02** \| _ \| **0.91 (0.84-0.99) 0.02** \| **0.92 (0.85-1) 0.04** \| _ \| \| KIRC \| _ \| _ \| _ \| **0.87 (0.75-1) 0.05** \| **0.84 (0.73-0.97) 0.02** \| _ \| \| LIHC \| **0.88 (0.82-0.95) 0** \| **0.88 (0.82-0.95) 0** \| _ \| **0.91 (0.85-0.96) 0** \| **0.89 (0.84-0.95) 0** \| **0.88 (0.82-0.96) 0** \| \| MESO \| **0.75 (0.58-0.96) 0.02** \| **0.75 (0.58-0.96) 0.02** \| **0.54 (0.37-0.78) 0** \| **0.72 (0.54-0.98) 0.04** \| **0.72 (0.54-0.97) 0.03** \| **0.66 (0.47-0.92) 0.01** \| \| SKCM \| **0.89 (0.81-0.98) 0.01** \| **0.9 (0.82-0.99) 0.02** \| _ \| _ \| _ \| _ \| \| UCEC \| **0.86 (0.79-0.93) 0** \| **0.87 (0.8-0.94) 0** \| _ \| **0.89 (0.83-0.96) 0** \| **0.89 (0.83-0.97) 0** \| _ \|   The result shown as hazard ratio ( 95% confidence interval ), statistical p-value.   \| **Table S3 Survival association with ESR2 mRNA expression using Cox regression** \| \| \| \| \| \| \| \| --- \| --- \| --- \| --- \| --- \| --- \| --- \| \| **Cancer** \| **Overall Survival** \| \| \| **Progression-Free Interval** \| \| \| \| **univariate COX** \| **multivariate COX (age/gender/race)** \| **multivariate COX (all)** \| **univariate COX** \| **multivariate COX (age/gender/race)** \| **multivariate COX (all)** \| \| BRCA \| **0.77 (0.65-0.92) 0** \| **0.81 (0.68-0.97) 0.02** \| **0.78 (0.64-0.97) 0.02** \| _ \| _ \| _ \| \| GBM \| _ \| _ \| _ \| **1.15 (1.01-1.31) 0.03** \| **1.14 (1-1.3) 0.05** \| _ \| \| KICH \| **2.19 (1.24-3.84) 0.01** \| **2.08 (1.2-3.61) 0.01** \| _ \| **2.17 (1.27-3.69) 0** \| **2.06 (1.2-3.53) 0.01** \| **2.68 (1.59-4.52) 0** \| \| KIRC \| **1.25 (1.13-1.39) 0** \| **1.26 (1.14-1.4) 0** \| _ \| **1.18 (1.06-1.33) 0** \| **1.22 (1.09-1.37) 0** \| _ \| \| KIRP \| **1.35 (1.04-1.75) 0.02** \| **1.34 (1.02-1.76) 0.04** \| **1.46 (1.12-1.9) 0.01** \| _ \| _ \| _ \| \| LGG \| **1.44 (1.14-1.82) 0** \| **1.43 (1.13-1.8) 0** \| **1.33 (1.07-1.64) 0.01** \| **1.19 (1-1.43) 0.05** \| **1.21 (1-1.45) 0.05** \| _ \| \| PAAD \| **0.78 (0.63-0.98) 0.03** \| **0.78 (0.63-0.98) 0.03** \| **0.7 (0.55-0.89) 0** \| _ \| _ \| _ \| \| STAD \| _ \| _ \| _ \| **1.22 (1.05-1.42) 0.01** \| **1.27 (1.09-1.49) 0** \| _ \| \| UVM \| **1.49 (1.05-2.12) 0.03** \| **1.46 (1.02-2.07) 0.04** \| _ \| **1.47 (1.08-2) 0.01** \| **1.46 (1.08-1.98) 0.02** \| _ \|   The result shown as hazard ratio ( 95% confidence interval ), statistical p-value.  **Table S4 Survival association with ESR1 methylation using Cox regression** | | | | | | |
| --- | --- | --- | --- | --- | --- | --- | --- | --- | --- | --- | --- | --- | --- | --- | --- | --- | --- | --- | --- | --- | --- | --- | --- | --- | --- | --- | --- | --- | --- | --- | --- | --- | --- | --- | --- | --- | --- | --- | --- | --- | --- | --- | --- | --- | --- | --- | --- | --- | --- | --- | --- | --- | --- | --- | --- | --- | --- | --- | --- | --- | --- | --- | --- | --- | --- | --- | --- | --- | --- | --- | --- | --- | --- | --- | --- | --- | --- | --- | --- | --- | --- | --- | --- | --- | --- | --- | --- | --- | --- | --- | --- | --- | --- | --- | --- | --- | --- | --- | --- | --- | --- | --- | --- | --- | --- | --- | --- | --- | --- | --- | --- | --- | --- | --- | --- | --- | --- | --- | --- | --- | --- | --- | --- | --- | --- | --- | --- | --- | --- | --- | --- | --- | --- | --- | --- | --- | --- | --- | --- | --- | --- | --- | --- | --- | --- | --- | --- | --- | --- | --- | --- | --- | --- | --- | --- | --- | --- | --- |
| **Cancer** | **Overall Survival** | | | **Progression-Free Interval** | | |
|  | **univariate COX** | **multivariate COX (age/gender/race)** | **multivariate COX (all)** | **univariate COX** | **multivariate COX (age/gender/race)** | **multivariate COX (all)** |
| BLCA | _ | _ | _ | **0.09 (0.02-0.43) 0** | **0.11 (0.02-0.56) 0.01** | _ |
| BRCA | **0.08 (0.01-0.49) 0.01** | **0.16 (0.03-0.99) 0.05** | _ | _ | _ | _ |
| KICH | _ | _ | _ | **4910544.36 (107.21-224916567536.4) 0** | **1479940.43 (24.55-89223727156.41) 0.01** | **66769512650.94 (4058258.96-1098541976945758) 0** |
| KIRC | _ | _ | _ | **153.59 (3.74-6299.75) 0.01** | **249.54 (5.36-11611.74) 0** | _ |
| KIRP | **238.62 (10.4-5476.87) 0** | **182.32 (7.26-4577.82) 0** | _ | **23.5 (1.14-482.83) 0.04** | **32.27 (1.52-683.89) 0.03** | _ |
| LAML | **0 (0-0.03) 0** | **0 (0-0.14) 0** | _ | _ | _ | _ |
| LGG | **0 (0-0.02) 0** | **0 (0-0.01) 0** | **0 (0-0.06) 0** | **0 (0-0.01) 0** | **0 (0-0.01) 0** | **0.01 (0-0.07) 0** |
| PCPG | _ | _ | _ | **38.52 (1.51-982.85) 0.03** | **64.24 (2.25-1832.62) 0.01** | _ |
| STAD | **0.12 (0.02-0.54) 0.01** | **0.17 (0.03-0.87) 0.03** | **0.12 (0.02-0.87) 0.04** | _ | _ | _ |

Result shown as: hazard ratio, ( 95% confidence interval ), statistical p-value.

| **Table S5 Survival association with ESR1 methylation using Cox regression** | | | | | | |
| --- | --- | --- | --- | --- | --- | --- |
| **Cancer** | **Overall Survival** | | | **Progression-Free Interval** | | |
|  | **univariate COX** | **multivariate COX (age/gender/race)** | **multivariate COX (all)** | **univariate COX** | **multivariate COX (age/gender/race)** | **multivariate COX (all)** |
| KIRP | **2002.75 (65.6-61142.45) 0** | **2636.57 (77.41-89805.72) 0** | **82.71 (1.35-5064.5) 0.04** | **1076.48 (34.42-33666.43) 0** | **1392.6 (44.75-43341.05) 0** | **68.96 (1.47-3242.57) 0.03** |
| LGG | **0 (0-0.01) 0** | **0 (0-0.01) 0** | **0.01 (0-0.05) 0** | **0 (0-0.01) 0** | **0 (0-0.01) 0** | **0.01 (0-0.05) 0** |
| PAAD | **0 (0-0.1) 0.01** | **0 (0-0.1) 0.01** | _ | **0 (0-0.48) 0.03** | **0 (0-0.44) 0.03** | _ |
| STAD | **0.04 (0-0.46) 0.01** | **0.04 (0-0.61) 0.02** | **0.01 (0-0.22) 0** | _ | _ | _ |
| UVM | **0 (0-0) 0.01** | **0 (0-0) 0.01** | _ | _ | _ | _ |

Result shown as: hazard ratio, ( 95% confidence interval ), statistical p-value.

| **Table S6 Survival association with ERα protein expression using Cox regression** | | | | | | |
| --- | --- | --- | --- | --- | --- | --- |
| **Cancer** | **Overall Survival** | | | **Progression-Free Interval** | | |
|  | **univariate COX** | **multivariate COX (age/gender/race)** | **multivariate COX (all)** | **univariate COX** | **multivariate COX (age/gender/race)** | **multivariate COX (all)** |
| KIRC | **0.54 (0.34-0.86) 0.01** | **0.51 (0.32-0.82) 0.01** | _ | **0.31 (0.18-0.52) 0** | **0.29 (0.17-0.5) 0** | _ |
| KIRP | **0.37 (0.15-0.91) 0.03** | **0.32 (0.13-0.83) 0.02** | _ | _ | _ | _ |
| LGG | **0.27 (0.15-0.47) 0** | **0.22 (0.12-0.4) 0** | **0.27 (0.15-0.49) 0** | **0.37 (0.23-0.58) 0** | **0.38 (0.24-0.6) 0** | **0.37 (0.24-0.57) 0** |
| MESO | **0.74 (0.56-0.99) 0.04** | **0.66 (0.47-0.92) 0.01** | **0.48 (0.28-0.81) 0.01** | **0.63 (0.44-0.92) 0.02** | **0.66 (0.45-0.97) 0.03** | _ |
| PRAD | _ | _ | _ | **2.18 (1.3-3.64) 0** | **2.15 (1.26-3.64) 0** | _ |
| UCEC | **0.74 (0.64-0.86) 0** | **0.74 (0.64-0.86) 0** | _ | **0.79 (0.69-0.9) 0** | **0.79 (0.7-0.9) 0** | **0.82 (0.69-0.99) 0.04** |

Result shown as: hazard ratio, ( 95% confidence interval ), statistical p-value.

| **Table S7 Survival association with Erα-pS118 protein expression using Cox regression** | | | | | | |
| --- | --- | --- | --- | --- | --- | --- |
| **Cancer** | **Overall Survival** | | | **Progression-Free Interval** | | |
|  | **univariate COX** | **multivariate COX (age/gender/race)** | **multivariate COX (all)** | **univariate COX** | **multivariate COX (age/gender/race)** | **multivariate COX (all)** |
| BLCA | **0.46 (0.29-0.71) 0** | **0.53 (0.32-0.89) 0.02** | **0.58 (0.34-1) 0.05** | _ | _ | _ |
| CESC | **0.13 (0.03-0.71) 0.02** | **0.12 (0.02-0.66) 0.01** | _ | _ | _ | _ |
| HNSC | **2.33 (1.05-5.17) 0.04** | **2.43 (1.09-5.41) 0.03** | _ | **3.52 (1.48-8.37) 0** | **3.83 (1.62-9.1) 0** | _ |
| KICH | _ | _ | _ | **0.55 (0.32-0.95) 0.03** | **0.47 (0.25-0.86) 0.02** | **0.4 (0.2-0.77) 0.01** |
| KIRP | **0.02 (0-0.18) 0** | **0.01 (0-0.12) 0** | **0.03 (0-0.63) 0.02** | **0.03 (0-0.21) 0** | **0.02 (0-0.15) 0** | **0 (0-0.07) 0** |
| UCEC | **0.5 (0.33-0.75) 0** | **0.52 (0.34-0.78) 0** | _ | **0.59 (0.42-0.84) 0** | **0.6 (0.43-0.86) 0** | _ |

Result shown as: hazard ratio, ( 95% confidence interval ), statistical p-value.

**Supplementary Figure S1-S12**

**Figure S1** **Expression of estrogen receptors between matched samples in TCGA cancers.**

(A) ESR1 mRNA expression between matched tumor and normal in different cancer types. (B) ESR2 mRNA expression between matched tumor and normal samples in different cancer types. Stars represent statistically significant differences. *: p value < 0.05; **: p value < 0.01; ***: p value < 0.001.


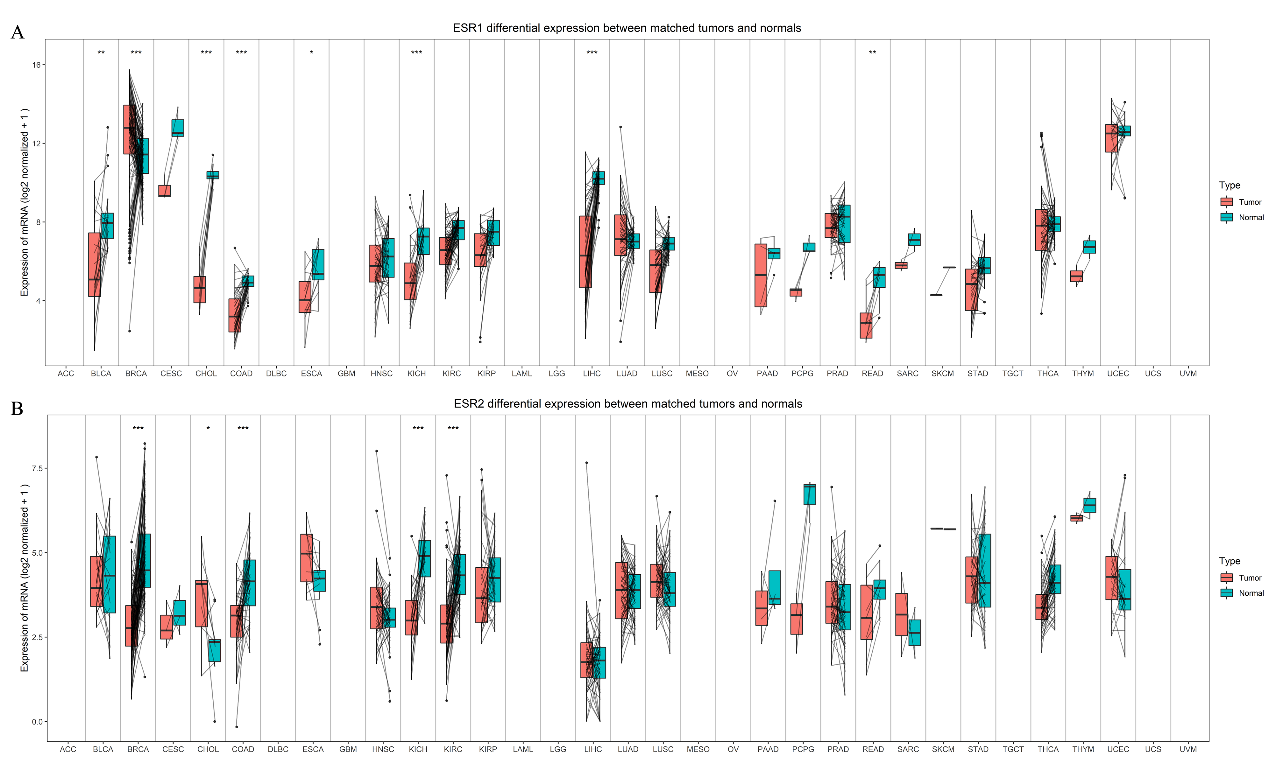


**Figure S2 The correlation between mRNA and methylation.**

The scatter plot showed the correlation between mRNA expression and DNA methylation of ESR1(top) and ESR2 (bottom) in each TCGA cancers. Significant correlation was marked by red star.
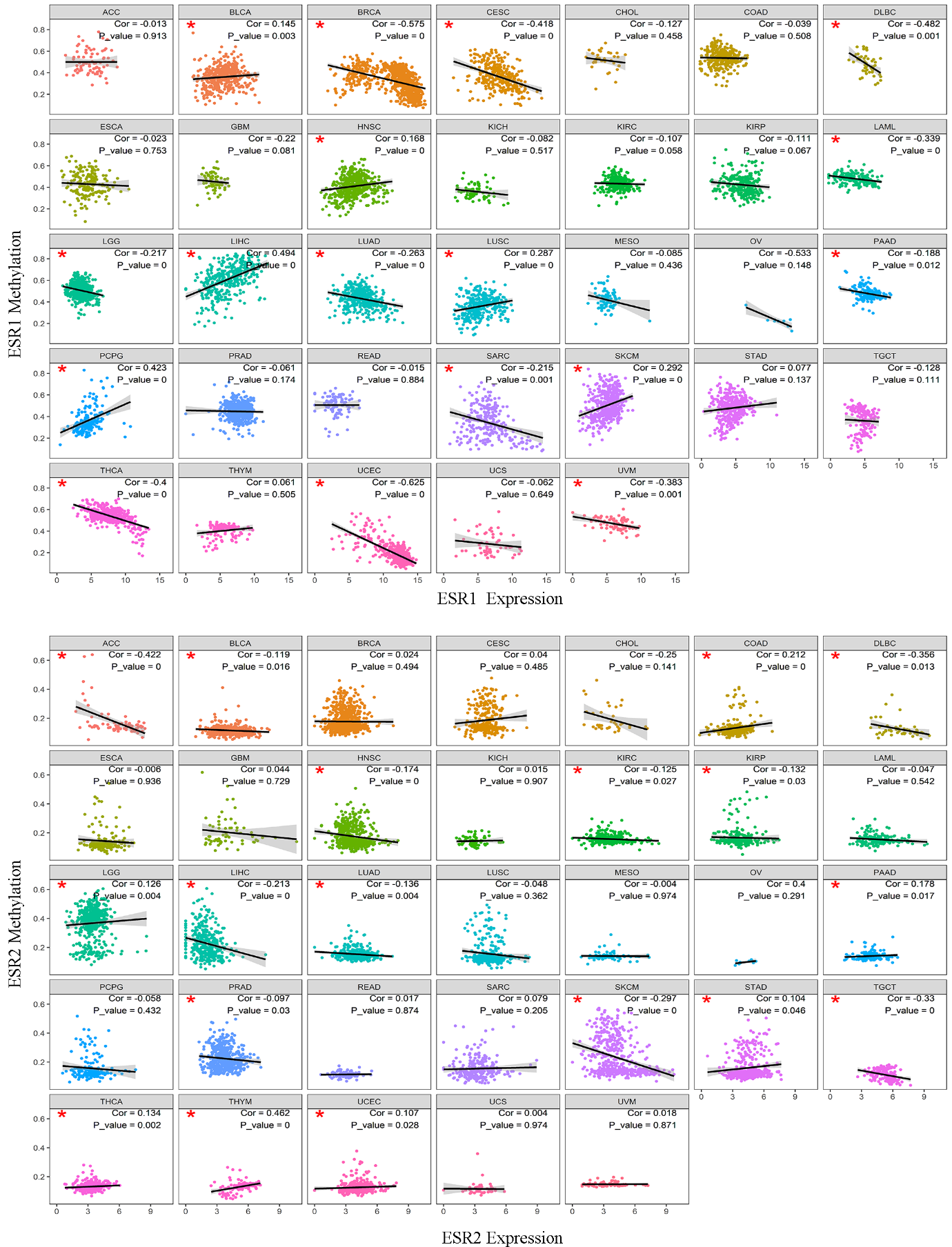


**Figure S3 The expression and correlation between different sex hormone receptors.**

The boxplot showed the expression of AR, ESR1, ESR2, PGR in different cancers (A); The heatmap showed the correlation among four hormone receptors in each cancers (B).

**
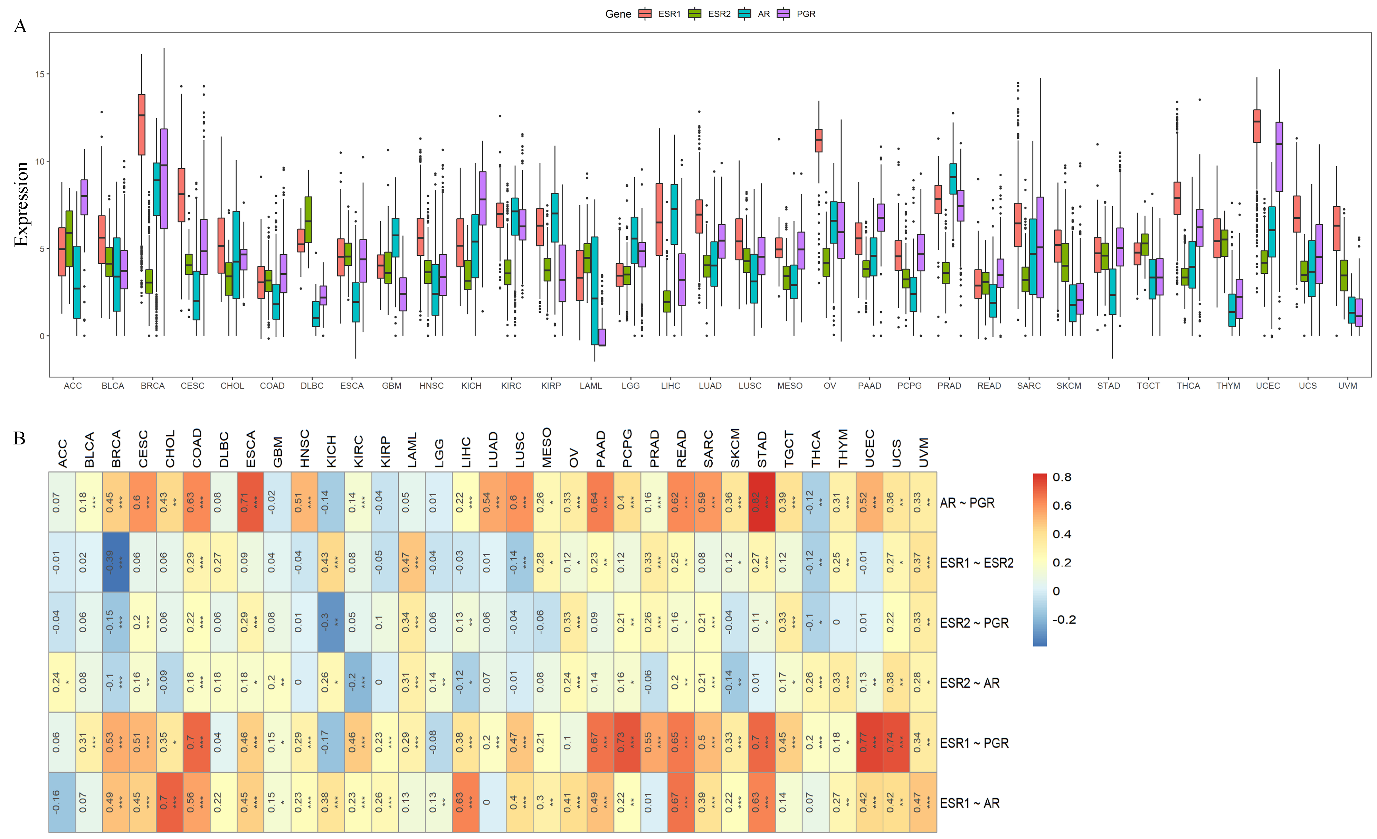
**

**Figure S4 The estrogen receptor correlated tumor subtype genes in ACC and THCA.**

The correlation between ESR1 expression and tissue-specific genes in ACC and THCA (left); and correlation between ESR1 or ESR2 expression and CLDN7 in ACC and THCA (right).
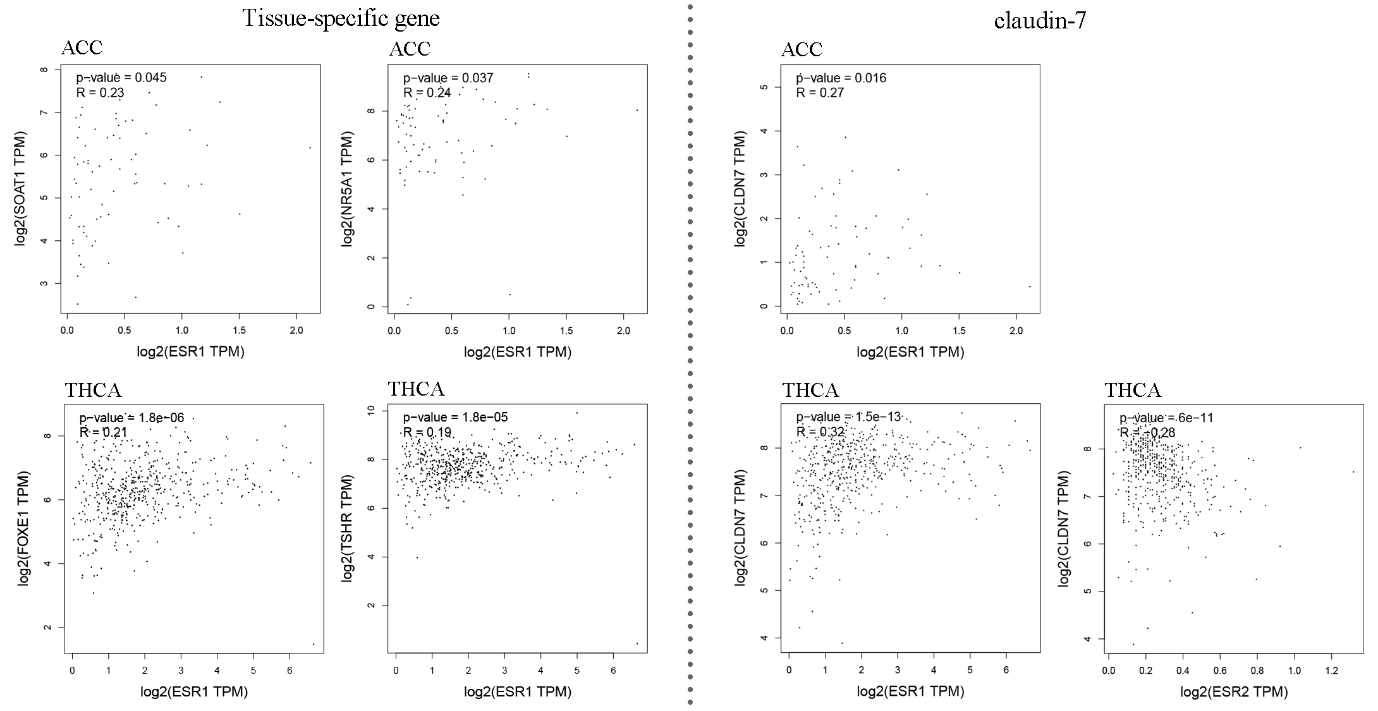


**Figure S5 The correlation between estrogen receptor and proliferation related genes.**

The correlation between ESR1 expression and proliferative phenotypes in LIHC (A); mitotic phenotype genes in UCEC (B); and cell cycle genes in each TCGA cancer (C).

**
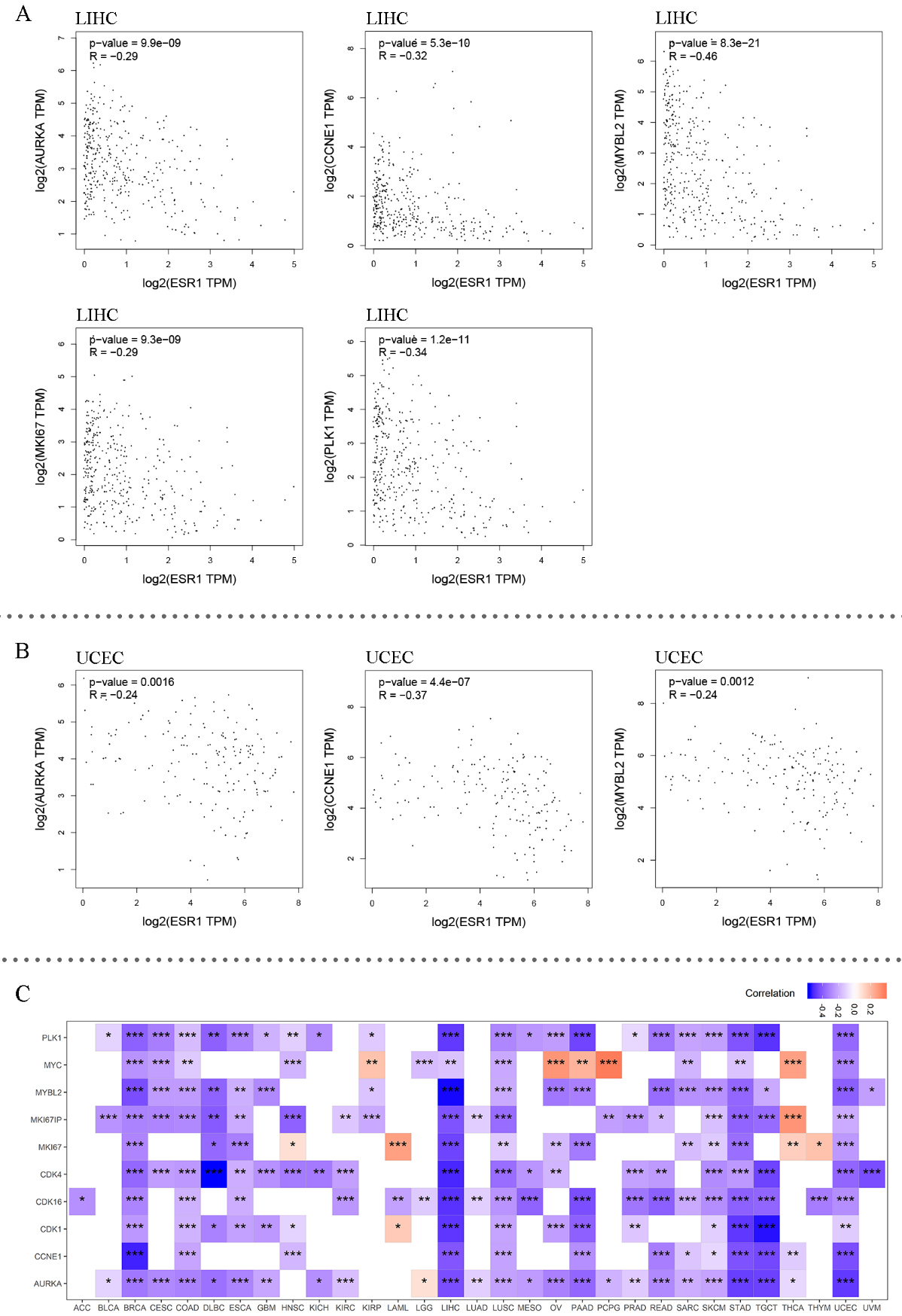
**

**Figure S6 The ESR1 expression between IDH1 wild type and IDH1 mutated tumors, GBM, LGG, LIHC and PRAD.**

**
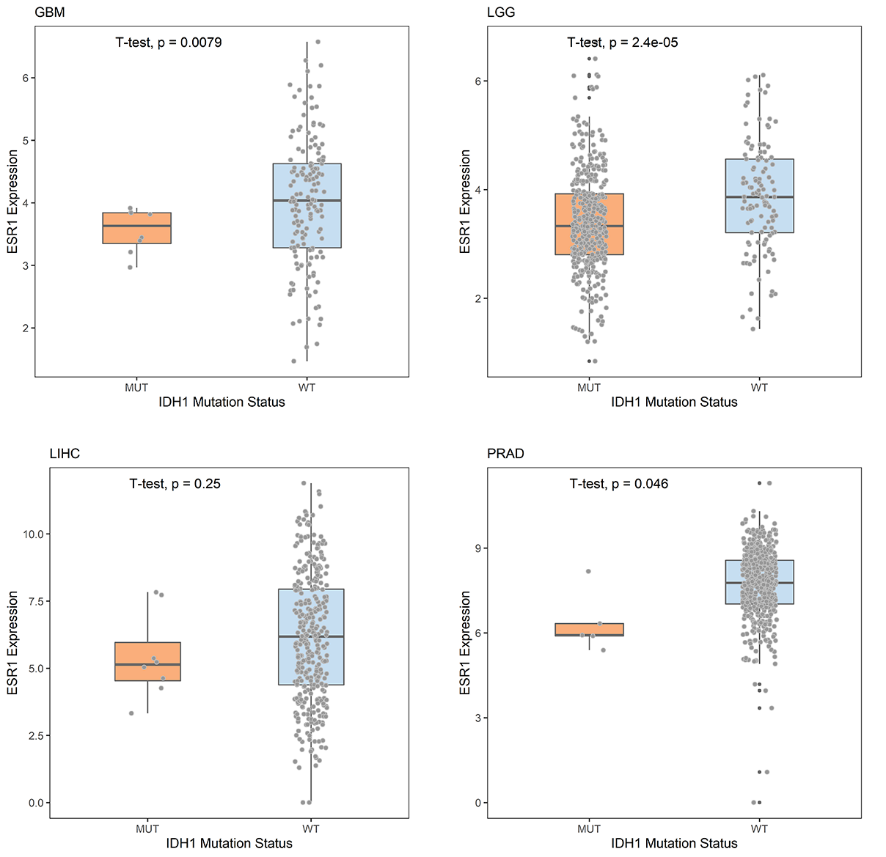
**

**Figure S7 The significant association of ESR1 mRNA expression with clinical characteristics in TCGA cancers.**

The expression of ESR1 is significant different across different clinical characteristics such as (A)gender, (B)age, (C)tumor status, (D)race, (E)grade, (F)stage. **
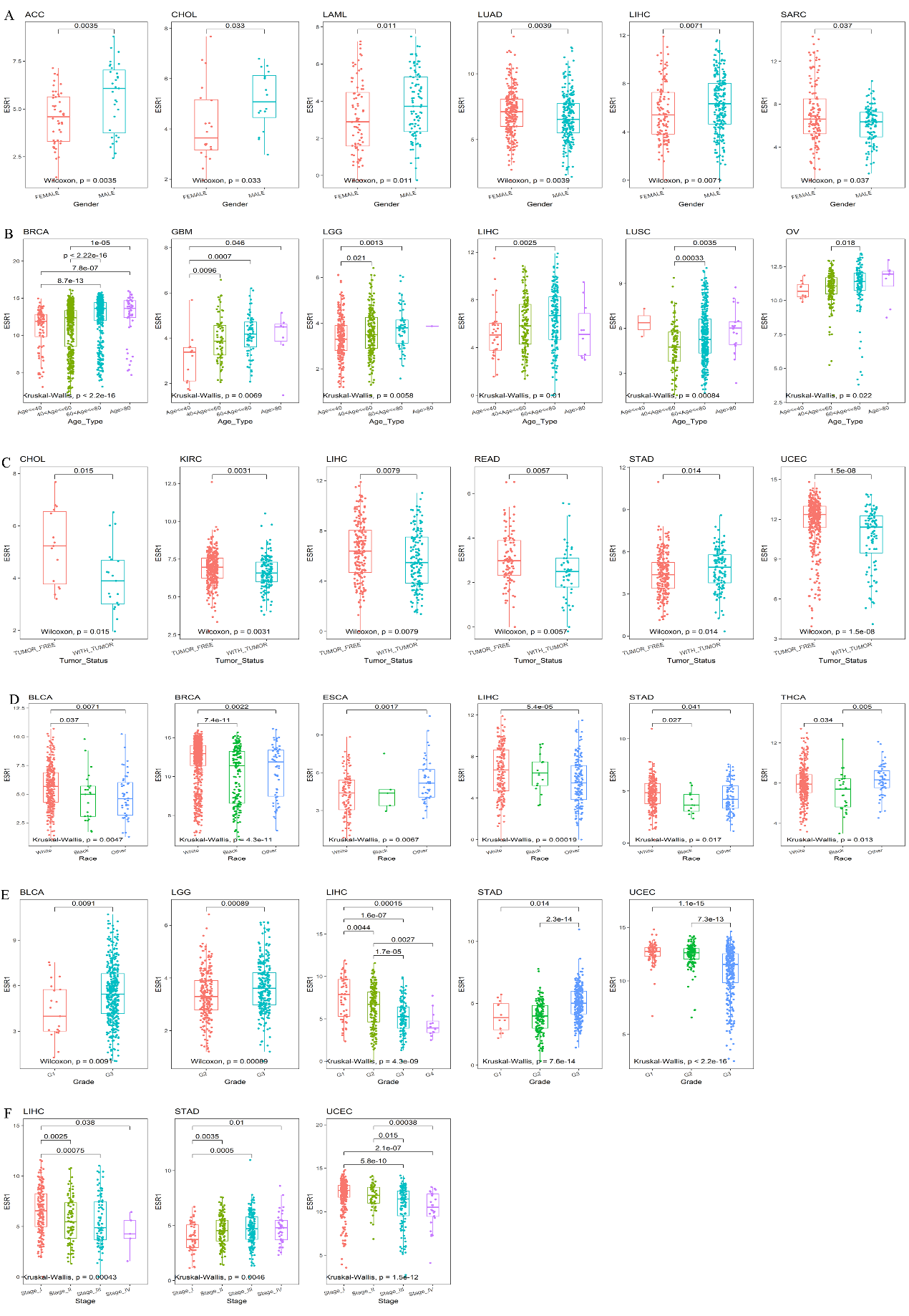
**

**Figure S8 The significant association of ESR2 mRNA expression with clinical characteristics in TCGA cancers.**

The expression of ESR2 is significant different across different clinical characteristics such as (A)gender, (B)age, (C)tumor status, (D)race, (E)stage.


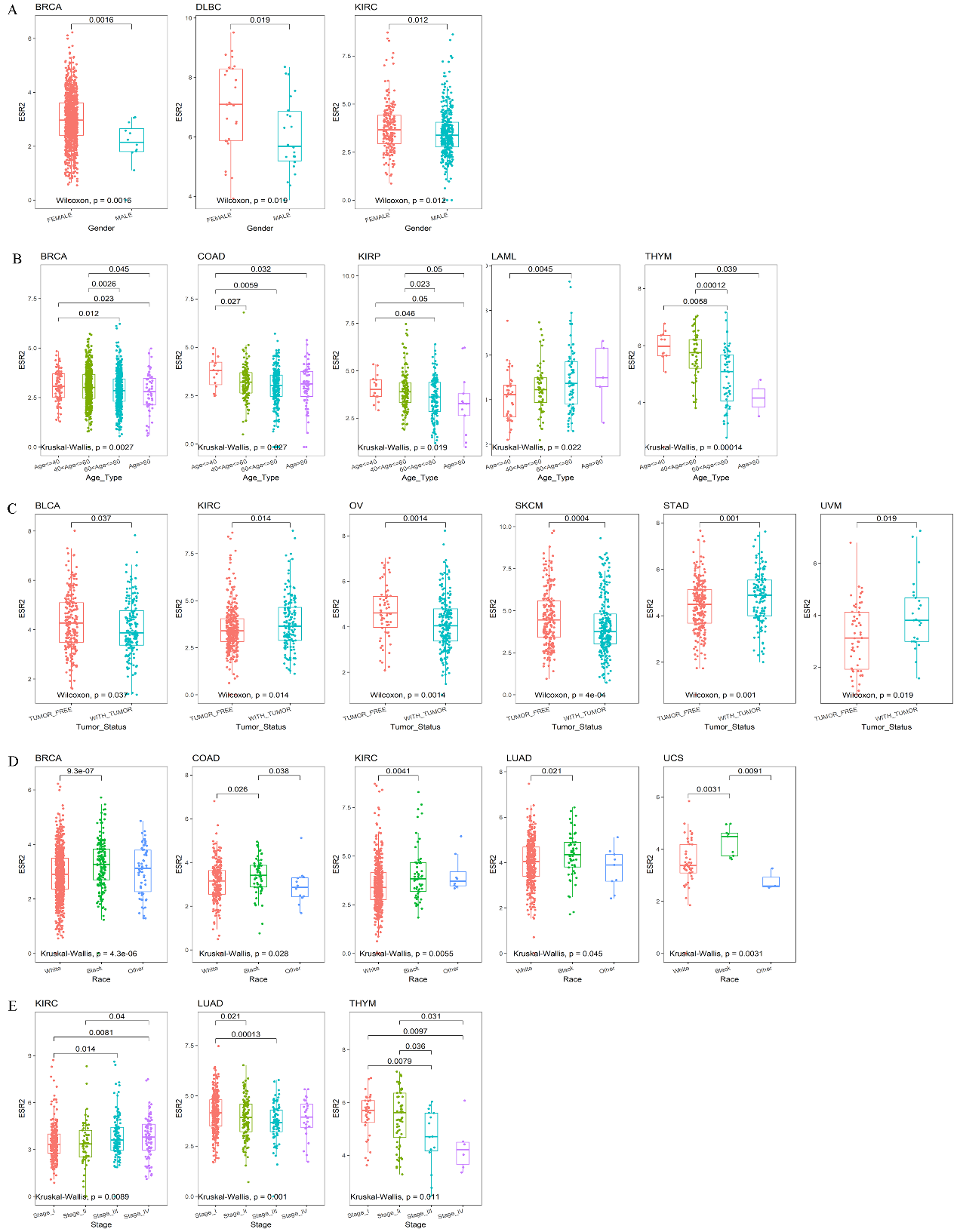


**Figure S9 The association of promoter methylation with survival of patients.**

Statistically significant survival difference (log rank p-value < 0.05) between high and low DNA methylation group were found in (A)BLCA, (B)BRCA, (C)LAML, (D)KIRC, (E)KIRP, (F)LGG, (G)STAD in ESR1; and (H)LGG, (I)STAD in ESR2.


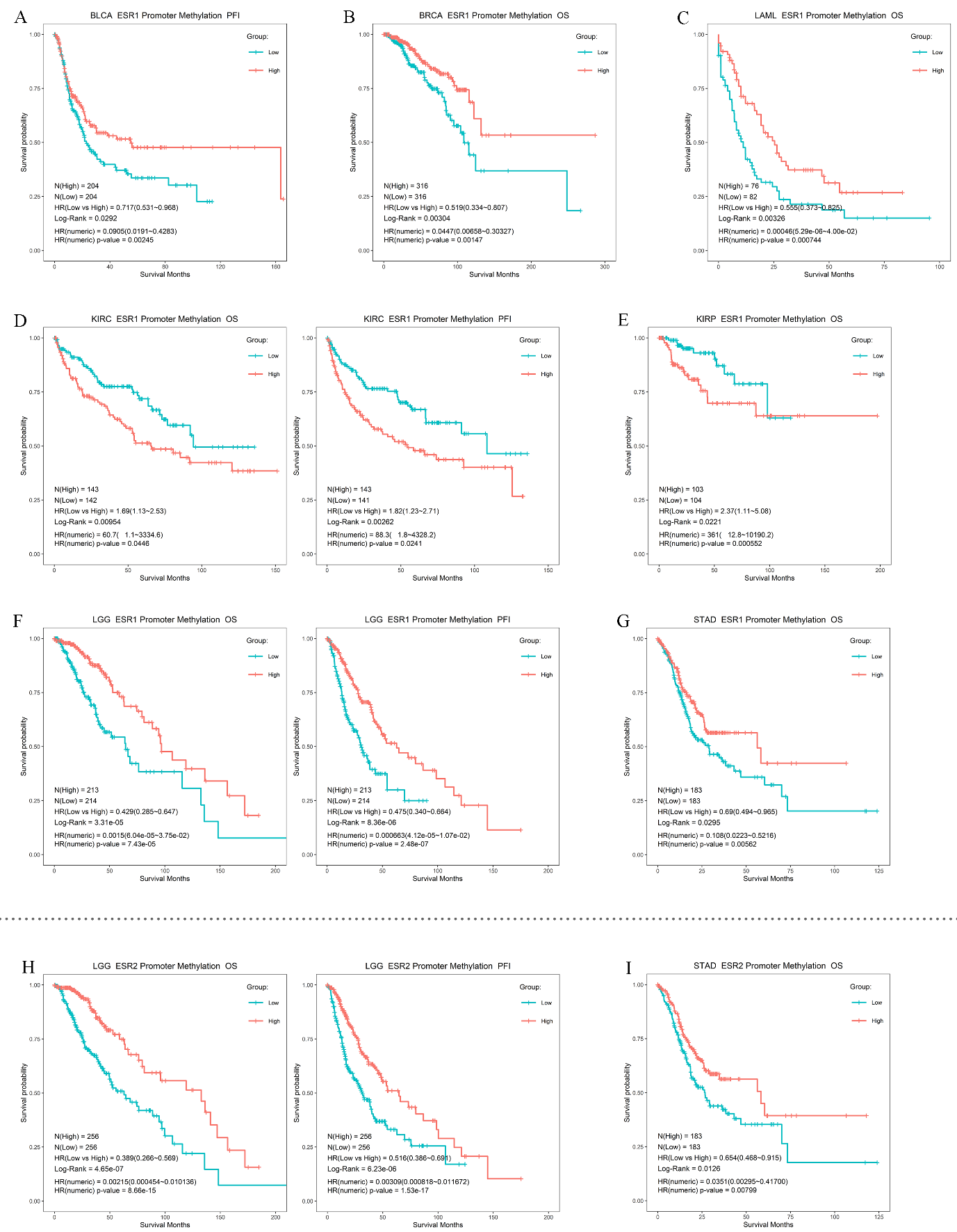


**Figure S10 The association of ERα protein expression with survival of patients in TCGA cancers.**

Statistically significant survival difference (log rank p-value < 0.05) between high and low ERα group were found in (A)ACC, (B)KIRC, (C)KIRP, (D)LGG, (E)PRAD, (F)LIHC, (G)SKCM, (H)UCEC. **
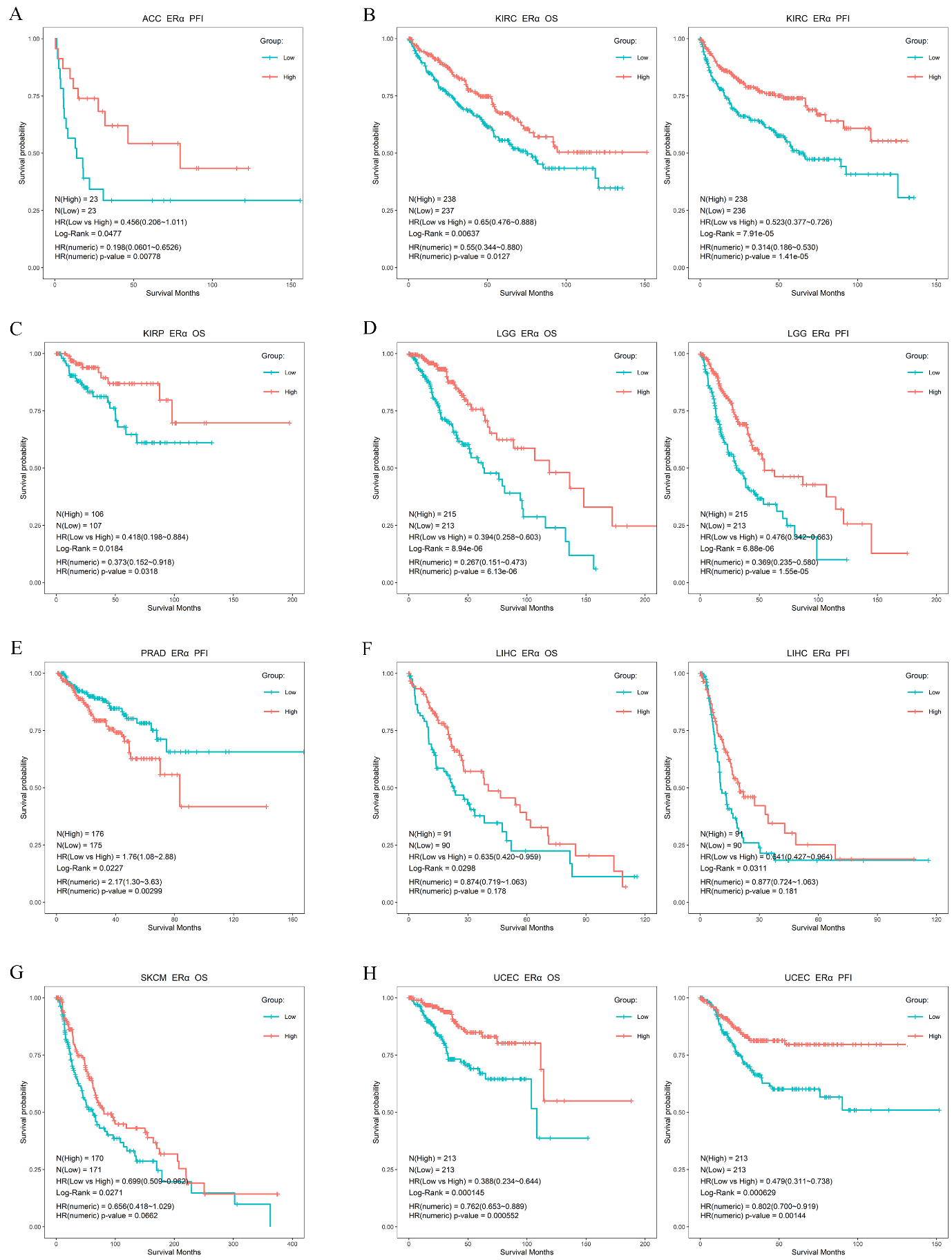
**

**Figure S11 The association of ERα-pS118 protein expression with survival of patients in TCGA cancers.**

Statistically significant survival difference (log rank p-value < 0.05) between high and low ERα-pS118 group were found in (A)BLCA, (B) HNSC, (C)KIRP, (D)KICH, (E)UCEC.


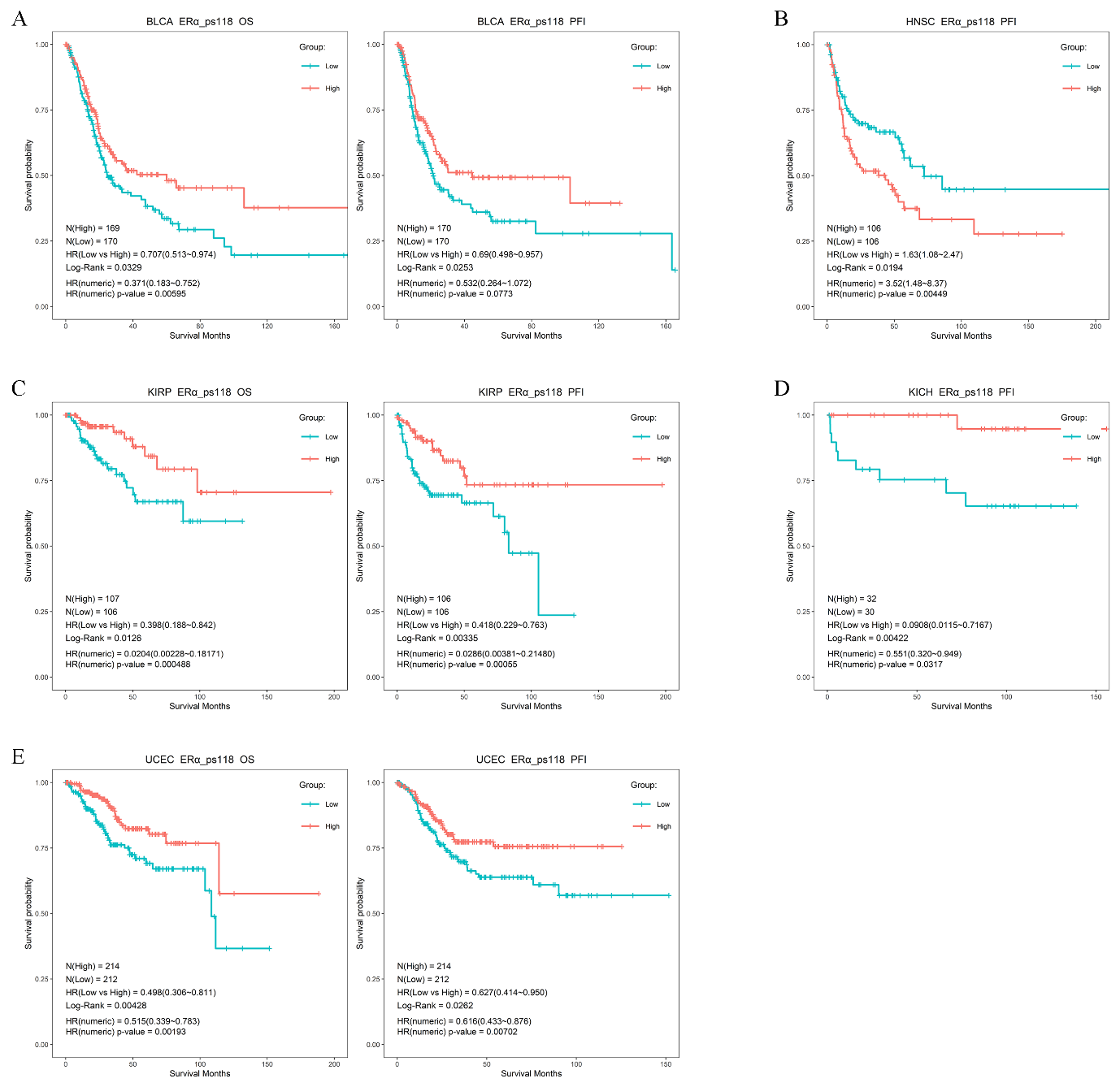


**Figure S12 estrogen receptors correlated genes and pathways GO biological process enrichment analysis.**

(A) Enriched GO biological process pathways of ESR1 correlated genes. (B) Enriched GO biological process pathways of ESR2 ­correlated genes.

**
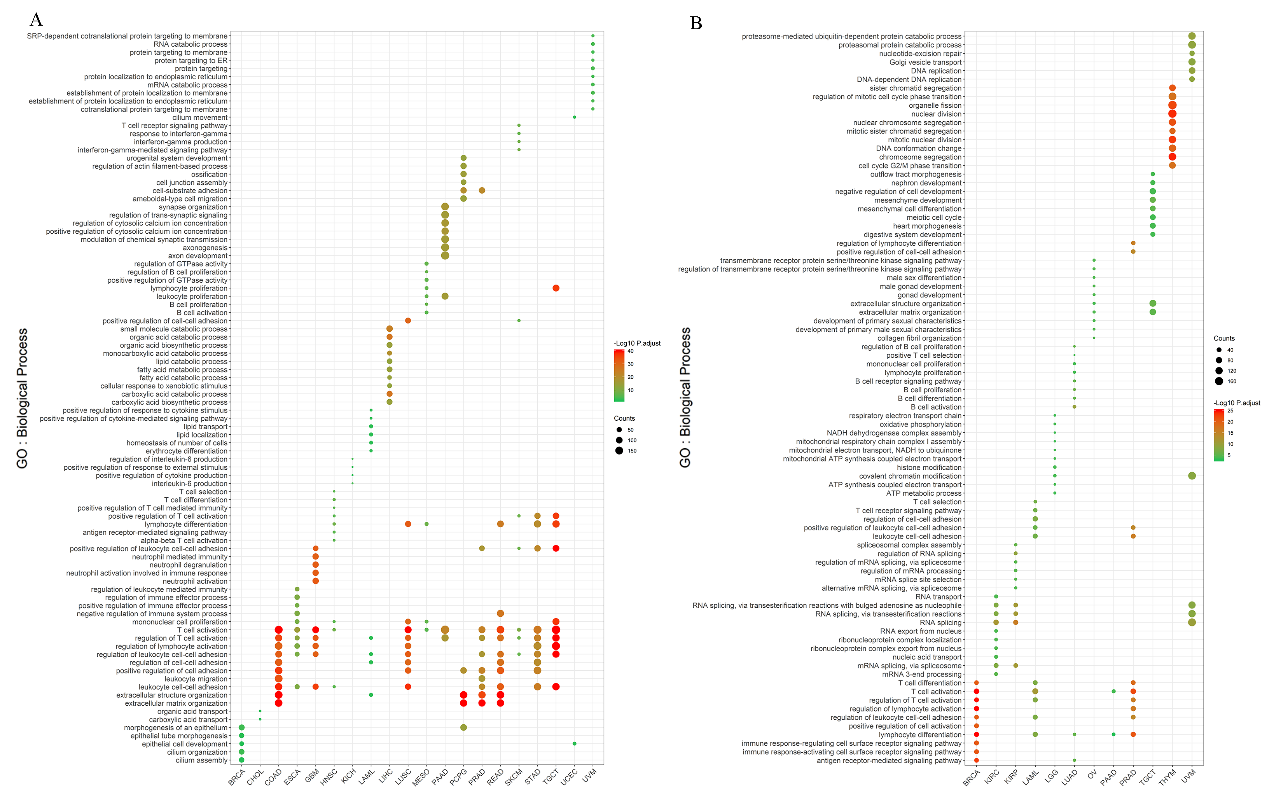
**

**Funding Information:**

This work was supported by grants from the National Young 1000 Talents Program of China, Jiangsu Province Education Department Grant, Jiangsu Province "Innovative and Entrepreneurial Team" and "Innovative and Entrepreneurial Talent" Grant, Chinese Society of Clinical Oncology Research Foundation and Southeast University-Nanjing Medical University Cooperative Research Project.
